# Densification of the interlayer spacing governs the nanomechanical properties of calcium-silicate-hydrate

**DOI:** 10.1038/s41598-017-11146-8

**Published:** 2017-09-08

**Authors:** Guoqing Geng, Rupert J. Myers, Mohammad Javad Abdolhosseini Qomi, Paulo J. M. Monteiro

**Affiliations:** 1 Department of Civil and Environmental Engineering, University of California, Berkeley, California 94720 United States; 2 School of Forestry & Environmental Studies, Yale University, New Haven, Connecticut 06511 United States; 3 Department of Civil and Environmental Engineering, University of California, Irvine, California 92697 United States; 4 Material Science Division, Lawrence Berkeley National Laboratory, Berkeley, California 94720 United States

## Abstract

Calciuam-silicate-hydrate (C-S-H) is the principal binding phase in modern concrete. Molecular simulations imply that its nanoscale stiffness is ‘defect-driven’, i.e., dominated by crystallographic defects such as bridging site vacancies in its silicate chains. However, experimental validation of this result is difficult due to the hierarchically porous nature of C-S-H down to nanometers. Here, we integrate high pressure X-ray diffraction and atomistic simulations to correlate the anisotropic deformation of nanocrystalline C-S-H to its atomic-scale structure, which is changed by varying the Ca-to-Si molar ratio. Contrary to the ‘defect-driven’ hypothesis, we clearly observe stiffening of C-S-H with increasing Ca/Si in the range 0.8 ≤ Ca/Si ≤ 1.3, despite increasing numbers of vacancies in its silicate chains. The deformation of these chains along the *b*-axis occurs mainly through tilting of the Si-O-Si dihedral angle rather than shortening of the Si-O bond, and consequently there is no correlation between the incompressibilities of the *a-* and *b-*axes and the Ca/Si. On the contrary, the intrinsic stiffness of C-S-H solid is inversely correlated with the thickness of its interlayer space. This work provides direct experimental evidence to conduct more realistic modelling of C-S-H-based cementitious material.

## Introduction

Over 4 billion metric tons of Portland cement (PC) was consumed in 2014, making PC concrete the dominant building material now and in the foreseeable future^1, 2^. A century of research into the performance of PC concrete has highlighted that optimizing the properties of the main ‘glue’-like, strength-giving phase in this material, calcium silicate hydrate (C-S-H), is a viable route to developing concrete with better performance and durability, and a lower carbon footprint^3–8^. C-S-H exists as a solid solution with variable structure and chemical composition over the Ca/Si molar ratio (Ca/Si) range from ~0.7 to ~1.6^3, 4^. It is also poorly crystalline and hierarchically porous at multiple length scales^5–8^. Such characteristics make it exceedingly difficult to probe its composition-structure-mechanical property relationships, particularly using laboratory-based equipment^3–9^, yet such information is fundamentally important in predicting and optimizing PC concrete performance through a ‘bottom-up’ simulation approach^10^.

**Figure 1:**
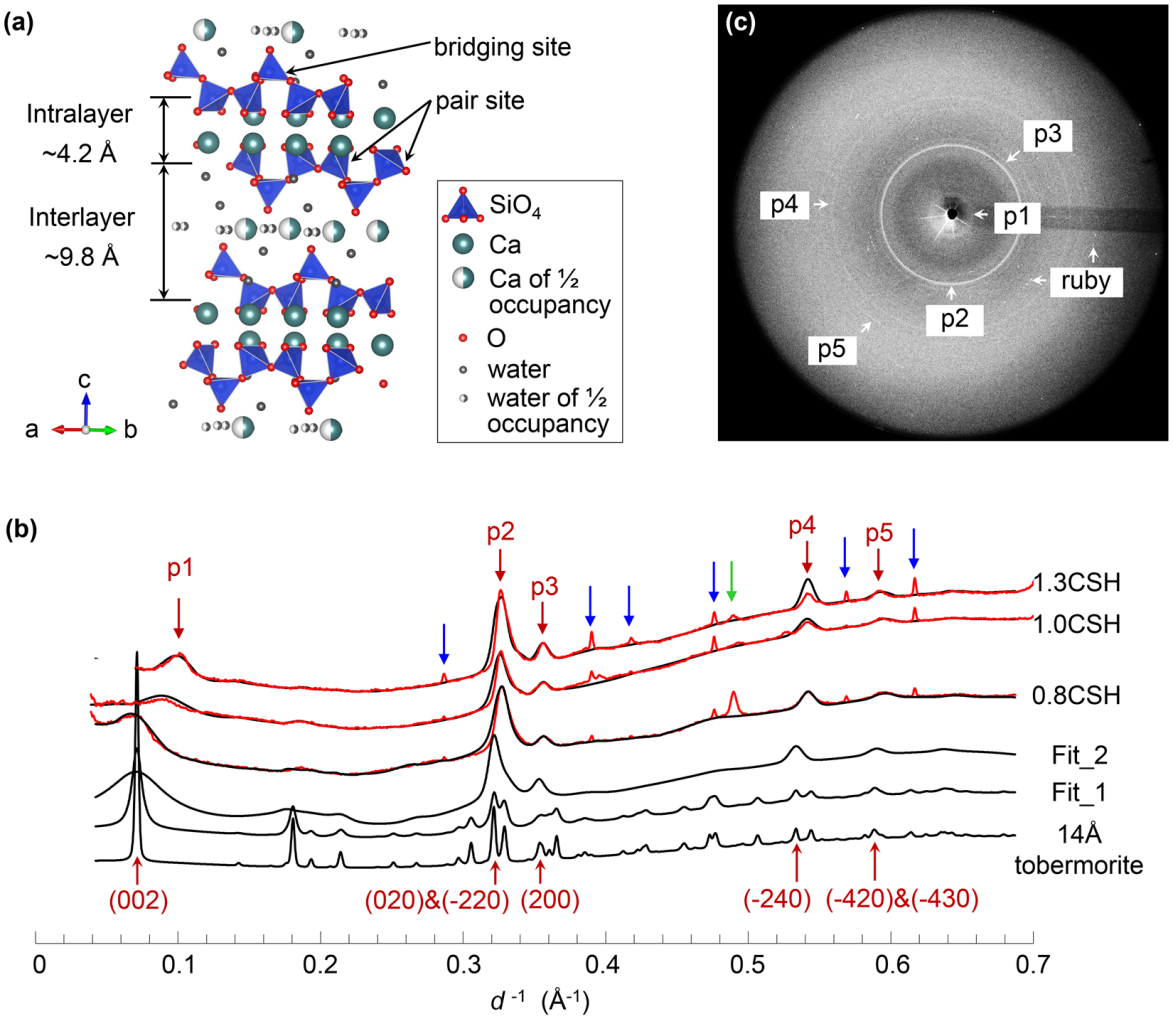
Refining the lattice parameters of C-S-H samples. (**a**) Crystal structure of 14 Å tobermorite viewed along the (110) direction^16^. The 14 Å-wide basal spacing comprises the intralayer (~4.2 Å) and interlayer (~9.8 Å) spacing. (**b**) 2D raw diffraction pattern of 0.8 CSH taken under ambient conditions. (**c**) Integrated diffractograms of 0.8C-S-H, 1.0CSH and 1.3CSH at ambient pressure (red curves). Peaks for C-S-H, ruby and steel gasket are labelled by red, blue and green arrows, respectively. Fitted diffractograms are plotted in black by refining the 14 Å tobermorite structure step-by-step considering nanocrystallinity effects (Fit_1), anisotropic strain-broadening (Fit_2), and adjusting the lattice parameters a, b, c, and γ (plotted together with the experimental data).

At Ca/Si < 1.5, the structure of C-S-H is nanocrystalline and analogous to that of tobermorite, henceforward referred to as C-S-H(I)^5, 6^. The tobermorite mineral family is composed of infinitely long ‘dreierketten’-type silicate chains bonded onto either side of CaO_7_ double sheets (Fig. 1a). These calcium silicate layers are separated by an aqueous electrolyte containing ‘interlayer’ space^11^. The combined thicknesses of a calcium silicate layer and its associated interlayer region is defined as the basal spacing, which is typically observed as 9.3, 11.3, and 14.0 Å in tobermorite minerals^12–16^. C-S-H(I) can achieve higher Ca/Si by omissions of ‘bridging site’ tetrahedra from its silicate chains and by accommodating aqueous Ca cations in the interlayer space (Fig. 1a)^17–19^. Grid nanoindentation indirectly probes the elastic properties of pore-free C-S-H, yet it remains strictly limited to the characteristic length of the interaction volume (~1 µm)^20–24^. Atomistic modeling probes higher resolution correlations between the stiffness and atomistic configuration of C-S-H(I)^25–30^. However, recent modeling advances have only yielded partial agreement between computational results and the few scattered published experimental data^25–28^. These modeling efforts have motivated the ‘defect-driven’ hypothesis, which claims that both the structural order and mechanical properties of C-S-H decrease with increasing Ca/Si^26, 29, 30^.

Contrary to the widely accepted ‘defect-driven’ hypothesis, highly crystalline 14 Å tobermorite exhibits significantly lower modulus than poorly crystalline but denser C-S-H phases^30–32^. This discrepancy promotes an alternative hypothesis that the nanomechanical properties of C-S-H are preferentially density-driven. Recent ab initio calculations also suggest that the Ca/Si is not an ideal metric for the cohesion of C-S-H; instead, its total bond order density provides a better index^33, 34^. The potential influence of the interlayer density on the overall mechanical properties of C-S-H was also suggested in a recent high-pressure X-ray diffraction (HP-XRD) study of calcium aluminosilicate hydrate (C-A-S-H), although not clearly differentiated from cross-linking effects^35^. In an effort to obtain direct evidence of this relationship, here we use synchrotron-radiation-based HP-XRD to track the lattice parameters of C-S-H samples as functions of hydrostatic pressure up to ~10 GPa. The results are used to interpretate the influence of atomic structure changes induced by increasing Ca/Si on the axial incompressibilities and the overall bulk modulus of C-S-H. Molecular modeling is conducted to elucidate the mechanism(s) governing the anisotropic nanomechanical behavior of C-S-H.

## Results

### HP-XRD of C-S-H with Ca/Si ~ 0.8, 1.0 and 1.3

Nanocrystalline C-S-H samples with Ca/Si ~ 0.8, 1.0 and 1.3 (hereafter named 0.8CSH, 1.0CSH and 1.3CSH) were synthesized by mixing stoichiometric amounts of CaO and SiO_2_ in deionized water at a water-to-solid ratio of 45 for 182 days. Their mean (silicate) chain lengths (MCL) were characterized as ~19 (0.8CSH) to ~5 (1.0CSH) and ~2 (1.3CSH), using ^29^Si magic angle spinning nuclear magnetic resonance (MAS NMR)^36^. The HP-XRD experiments were conducted at beamline 12.2.2 of the Advanced Light Source (ALS), using an axial Merrell-Bassett cell. We used stainless steel as gaskets, ruby (Cr^3+^-doped Al_2_O_3_) as pressure calibrant, and methanol-ethanol solution (respective volumetric ratio of 4:1) as pressure medium. Sample preparation and HP-XRD experiment details are described in the methods section and the supporting information (SI). In the 2D raw diffraction image (Fig. 1b), C-S-H samples produces continuous diffraction rings (p1-p5), whereas ruby has intense and discrete diffraction spots. Other than the diffraction peaks of ruby and stainless steel gasket (Fig. 1c, blue and green arrows), the diffractograms (red curves in Fig. 1c) are highly comparable to the reported XRD of C-S-H(I), where most resolvable diffraction peaks (p2-p5) match the *hk*0 reflections of tobermorite^5, 37, 38^, except that peak p1 corresponds to the (002) diffraction of tobermorite. The diffraction rings from C-S-H are highly isotropic (i.e. no signs of texture), and do not overlap with diffractions of ruby or gasket.

It is well established that the diffuse diffraction peaks of C-S-H(I) can be fit using multiple tobermorite structures. The fitted lattice parameters refined using different structures are highly comparable^35, 37^. This is because of the poorly-crystalline nature of C-S-H that produces few diffuse peaks which only allows reliable fitting of the lattice parameters, but prohibits resolving any further information about its atom positions. Therefore, starting from structural models that are primarily composed of the tobermorite-type calcium silicate layer structure, a satisfactory fitting can always be obtained by considering a few key crystalline features of C-S-H^35^.

Here, we fit the experimental X-ray diffractograms of C-S-H using the 14 Å tobermorite structure in three steps: a) setting the crystallite size to 15 nm along each crystallographic axis (Fig. 1c, Fit_1); b) reducing the crystallite size along the *c*-axis from 15 nm to ~3–5 nm and introducing anisotropic strain-broadening effects^39^ to the *c*-direction (Fig. 1c, Fit_2); and c) refining the lattice parameters *a*, *b, c* and *γ*. We obtain a well-refined set of lattice parameters and a reasonable estimation of the crystallite sizes for each X-ray diffractogram fit; see SI for the details of refinement strategy, and the refined lattice parameters at ambient pressure. Refinement of the lattice parameter *c* relies on peak p1 (002), while the positions of the other labeled peaks p2-p5 depend greatly on the lattice parameters *a* and *b*. These results imply that C-S-H(I) is relatively poorly stacked along its *c*-axis, whereas the *ab-*plane is more ordered. Samples with different Ca/Si share similar *a* and *b* parameters at ambient pressure but distinct *c* values, which decrease with increasing Ca/Si.

**Figure 2:**
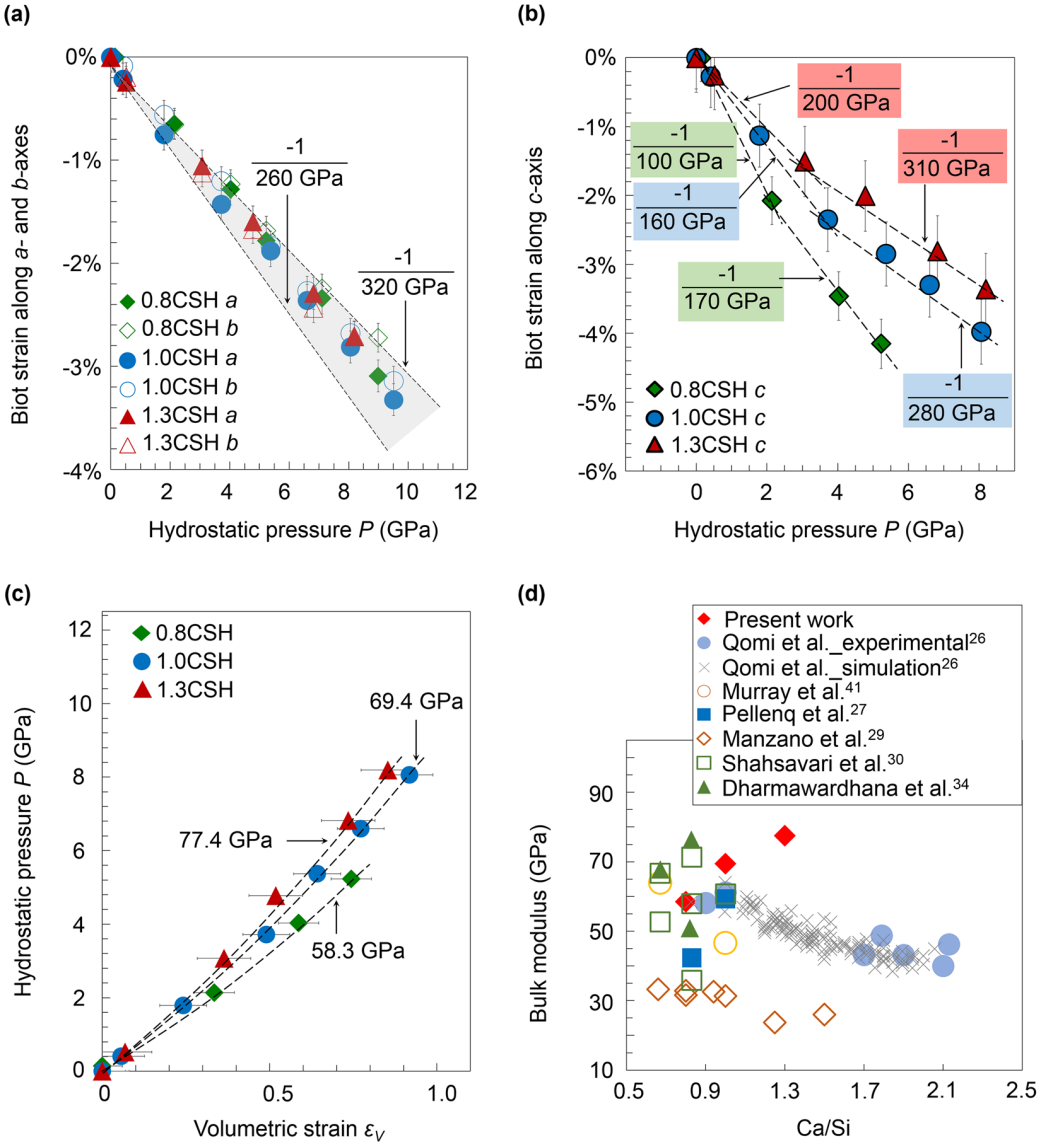
The mechanical response of C-S-H to hydrostatic pressure: Biot strain along a- and b-axes (**a**), and c-axis (**b**), are plotted as functions of the hydrostatic pressure. The slopes of the dashed lines (values indicated in the plots) are the incompressibility values, which is also the negative inverse of the stiffness along each direction. In (**b**), the c-axis of each sample can be fitted with two linear trendlines, with connection points at ~2, ~4 and ~3 GPa for 0.8CSH, 1.0CSH and 1.3CSH, respectively. (**c**) Fitting the second order Birch–Murnaghan equation of state (BM-EoS) yields the ambient bulk modulus *K*_0_; see SI. The average goodness of fit is 0.999 with standard error of approximately 1 GPa. (**d**) The bulk modulus of C-S-H plotted as a function of the Ca/Si measured experimentally and simulated using atomistic C-S-H models^26, 27, 29, 30, 41^. In (**d**), reported elastic moduli are recast as bulk modulus by assuming isotropic behavior with Poisson’s ratio of 0.25 when the bulk modulus is not directly available^26^.

To estimate the anisotropic incompressibilities of the C-S-H samples, we investigate the Biot strain along each lattice direction as functions of the hydrostatic pressure. We consider the lowest probed pressure for each sample to be the ambient condition in the following analysis, which is 0.14 GPa for 0.8CSH and atmospheric pressure for 1.0CSH and 1.3CSH. The complete set of diffractograms and refined lattice parameters are available in the SI. The Biot strain is calculated as (*l*−*l*_0_)/*l*_0_, where *l* and *l*_0_ are the measured and ambient pressure lattice parameters. The incompressibility is defined as the slope of the Biot strain versus pressure, which is, by definition, the negative inverse of stiffness. Despite notable differences in the defect content, the incompressibilities determined along the *a-* and *b-*axes of 0.8CSH, 1.0CSH, and 1.3CSH fall within the same range (−1/260 to −1/320 GPa), and are essentially independent of the Ca/Si (grey area in Fig. 2a). Biot strains along the *c*-axis of each sample can be fitted with two linear trendlines, with connection points at ~2, ~4 and ~3 GPa for 0.8CSH, 1.0CSH and 1.3CSH, respectively (Fig. 2b). In the low-pressure linear region, the *c-*axes of all samples are softer than their *a-* and *b-* axes, and exhibit an stiffening trend with increasing Ca/Si. In the high-pressure linear region, there is a 50–70% increase in c-axis stiffness compared to the ambient measurement. The *c-*axes of 1.0CSH and 1.3CSH exhibit respective incompressibilities of −1/280 and −1/310 GPa, which are comparable to those along their respective *a-* and *b-*axes. However, the *c*-axis in 0.8CSH remains significantly softer than its *ab*-plane throughout the whole pressure range.

By fitting the Birch–Murnaghan equation of state (BM-EoS^40^), the initial bulk modulus (*K*_*0*_) of 0.8C-S-H, 1.0C-S-H, and 1.3C-S-H are determined respectively as 58.3, 69.7, and 77.2 GPa (Fig. 2c). Compared with the existing data in Fig. 2(c) ^26, 27, 29, 30, 41^, our experimental results agree with some atomistic simulations and nanoindentation data at Ca/Si < 1. However at Ca/Si > 1, existing atomistic simulation studies suggest that increasing Ca/Si reduces the overall mechanical properties of C-S-H^26, 29, 41^, which is contrary to our experiment results. To further investigate the origin of this discrepancy between the HP-XRD results and the rest of the literature, we perform atomistic simulations using models that we develop and validate against the existing crystallographic and chemical information of the samples studied here. The direct coupling of experimental and model parameters distinguishes this work from other efforts published in the literature^26–30^.

### Insights into the mechanism of deformation using atomistic modelling

Here, model development begins with the structures of 14 Å tobermorite (for 0.8CSH and 1.0CSH) and 9 Å tobermorite (for 1.3CSH). We set the amount of bridging site vacancies and interlayer Ca to match the existing chemical composition and ^29^Si MAS NMR data^36^. The interlayer water content is then adjusted such that the basal spacing in the relaxed atomistic C-S-H model matches that determined by HP-XRD at ambient pressure. Note that we build 1.3CSH model from 9 Å tobermorite because its layer structure stacking pattern allows a relaxed basal spacing smaller than 10 Å, whereas other tobermorite models do not. Although the H_2_O-to-Si ratio (H/S) in 9 Å tobermorite structure is extremely low, the H/S in our 1.3CSH model is ~1.3, in consistency with reported values^19^. The initial configurations and atom positions are available in SI, along with a step-by-step development of the models. For the calculation, we use the GULP package^42^ and transferrable CSH-FF potential^43^. We incrementally increase the simulated hydrostatic pressure up to 8 GPa with intervals of 0.1 GPa. At each pressure, we quasi-statically minimize the enthalpy to find the updated atomic positions and cell dimensions, with a convergence norm tolerance of 10^−8^, while enforcing rational functional optimization below a norm of 10^−4^ to avoid unstable configurations.

**Figure 3:**
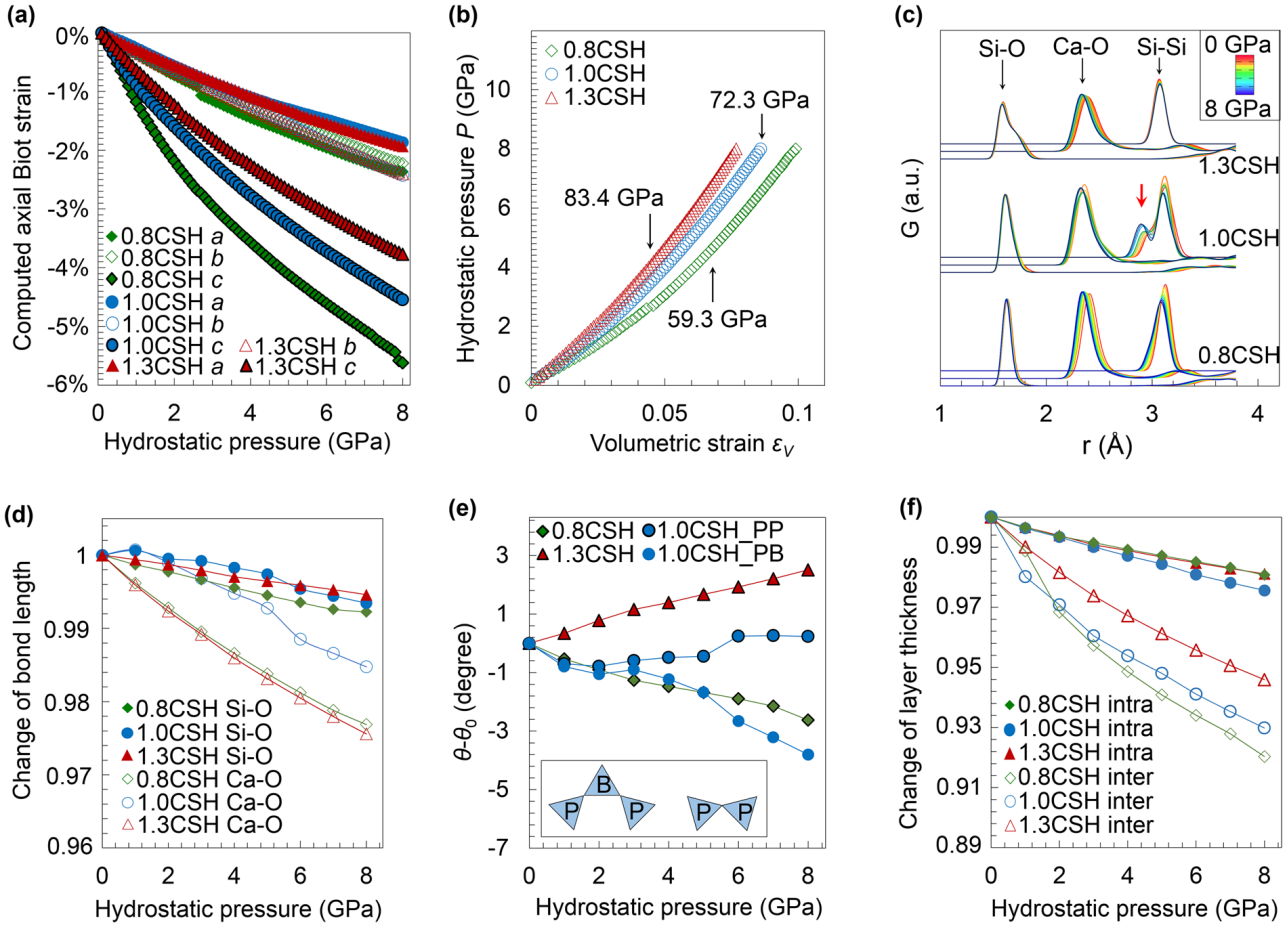
Atomistic simulations under hydrostatic pressure up to 8 GPa. (**a**) Biot strain of *a-, b-* and *c-*axes. The 1.3CSH model is modified from 9 Å tobermorite, and has a different unit cell shape to the 0.8CSH and 1.0CSH models which are constructed from 14 Å tobermorite. To allow direct comparisons among all models, the strain data of 1.3CSH are therefore recast by defining the *b*-axis to run parallel to its silicate dreierketten chains, the *a-*axis to be in-planar by 122.5° relative to the *b*-axis, and the *c*-axis to be perpendicular to the *ab*-plane. (**b**) Volumetric strain is plotted as a function of pressure; results of second order BM-EoS fitting are displayed. (**c**) Pair distribution functions (PDF) of Si-O, Ca-O and Si-Si pairs. Baselines of the curves are adjusted for viewing convenience only. The red arrow indicates the splitting of the first Si-Si peak. (**d**) Average Ca-O and Si-O bond deformation. (**e**) Si-O-Si dihedral angle change. The inset sketches non-defected Si chain units (left) and the dimeric structure obtained after removing silicate tetrahedra in bridging sites (right). P and B stand for pair and bridging sites, respectively. (**f**) The relative thicknesses of the interlayer and intralayer spacings of the atomistic C-S-H models compared to the ambient pressure values are plotted as functions of the applied hydrostatic pressure.

As shown in Fig. 3a, these model structures readily exhibit non-linearity and softness along the *c-*axis relative to the *a-* and *b-*axes. At 8 GPa, the computed Biot strains along the *a-* and *b*-axes in the atomistic simulations (−1.9% to −2.4%) are close to those measured experimentally (−2.2% to −2.8%), and the *c*-axis strains are fully consistent between simulation and experiment. These models not only exhibit *c*-axis stiffening with increasing Ca/Si, but also reproduce the pressure-induced stiffening along the *c*-axis. The BM-EoS approach yields ambient pressure bulk moduli of 59.3, 72.3 and 83.4 GPa, respectively for 0.8CSH, 1.0CSH and 1.3CSH (Fig. 3b), which closely match the experimental results. These results suggest that, by matching the experimental crystallographic information, these atomistic models are rational representations of the C-S-H molecular structure in our experiments. Therefore, we hereafter utilize them to probe the underlying mechanisms responsible for the observed relationships between the elastic properties of C-S-H and its Ca/Si.

We compute the pair distribution functions (PDF) of Si-O, Ca-O and Si-Si pairs across the entire range of simulated pressures (Fig. 3c), based on which the length change of Si-O and Ca-O bonds (Fig. 3d) and the dihedral angle change of Si-O-Si (Fig. 3e) are calculated. At 8 GPa, the Si-O pair near 1.62 Å linearly shortens by 0.4–0.6% relative to the ambient pressure case. The significant drifting of Ca-O peaks in the PDFs (Fig. 3c) correspond to bond shortening of ~2.4% for 0.8CSH and 1.3CSH, and ~1.2% for 1.0CSH, at 8 GPa relative to ambient pressure. The silicate chain is continuous in 0.8CSH and purely dimeric in 1.3CSH. In both cases, we observe a single Si-Si PDF peak as pressure increases. At 8 GPa, the Si-O-Si angle decreases by ~2.8° in 0.8CSH but increases by ~1.6° in 1.3CSH relative to the ambient pressure values, causing the closest Si-Si distance to decrease by ~1.6% and increase by ~0.2%, respectively. As shown in the inset of Fig. 3e, two distinct Si-Si linkages, i.e., pair-pair and bridging-pair, occur in 1.0CSH as a result of bridging site vacancies. They exhibit distinct angular distortion under pressure. The pair-pair dihedral angle (1.0CSH_PP) remains almost unchanged, whereas the pair-bridging dihedral angle decreases by ~3.8° at 8 GPa relative to its ambient pressure value. Therefore the continuous dreierketten chains deform mainly by the relative tilting of adjacent pair-bridging silicate tetrahedra instead of the shortening of the Si-O bond; the discontinuous silicate dimers do not significantly contribute to the deformation along the *b*-axis.

### Correlation between the basal spacing and bulk modulus of C-S-H phases

Both our experiments and simulations confirm that the major difference in mechanical response of the three samples is along the *c*-axis under hydrostatic pressure. Figure 3f shows the evolution of intralayer and interlayer spacing as functions of the hydrostatic pressure. The interlayer deforms much more readily than the intralayer in 0.8CSH and 1.0CSH, and dominates their overall deformation along the *c*-axis. When the silicate chains are entirely dimeric, i.e., in 1.3CSH, the much thinner and more Ca-enriched interlayer space becomes markedly stiffer, but is still softer than its intralayer. Therefore, the thinning and Ca-enriching of the interlayer space, caused by increasing the Ca/Si, controls the overall bulk modulus of C-S-H. The (non-cross-linked) silicate dreierketten chains do not provide the main resistance to compression, regardless of the amount of bridging site vacancies. These findings are in full agreement with the deformation mechanism of a broad range of zeolites with SiO_4_ frameworks, that the rotation of corner-sharing tetrahedra account for the majority of the deformation at relatively low pressures (e.g. <10 GPa), rather than contractions of Si-O bonds^44^. Compression occurs mainly through volumetric strain of cation-oxygen polyhedra (e.g., CaO_8_ octahedron in grossular^45^), compensated by tilting of SiO_4_ tetrahedra, such that the overall modulus depends mostly on the compressibility of cation-oxygen polyhedra^45, 46^. Ab-initio calculations on a broad range of crystalline calcium silicate hydrates also reveal that their moduli are positively correlated with the bond order density of Ca-O, but no direct correlation with that of Si-O^34^.

**Figure 4:**
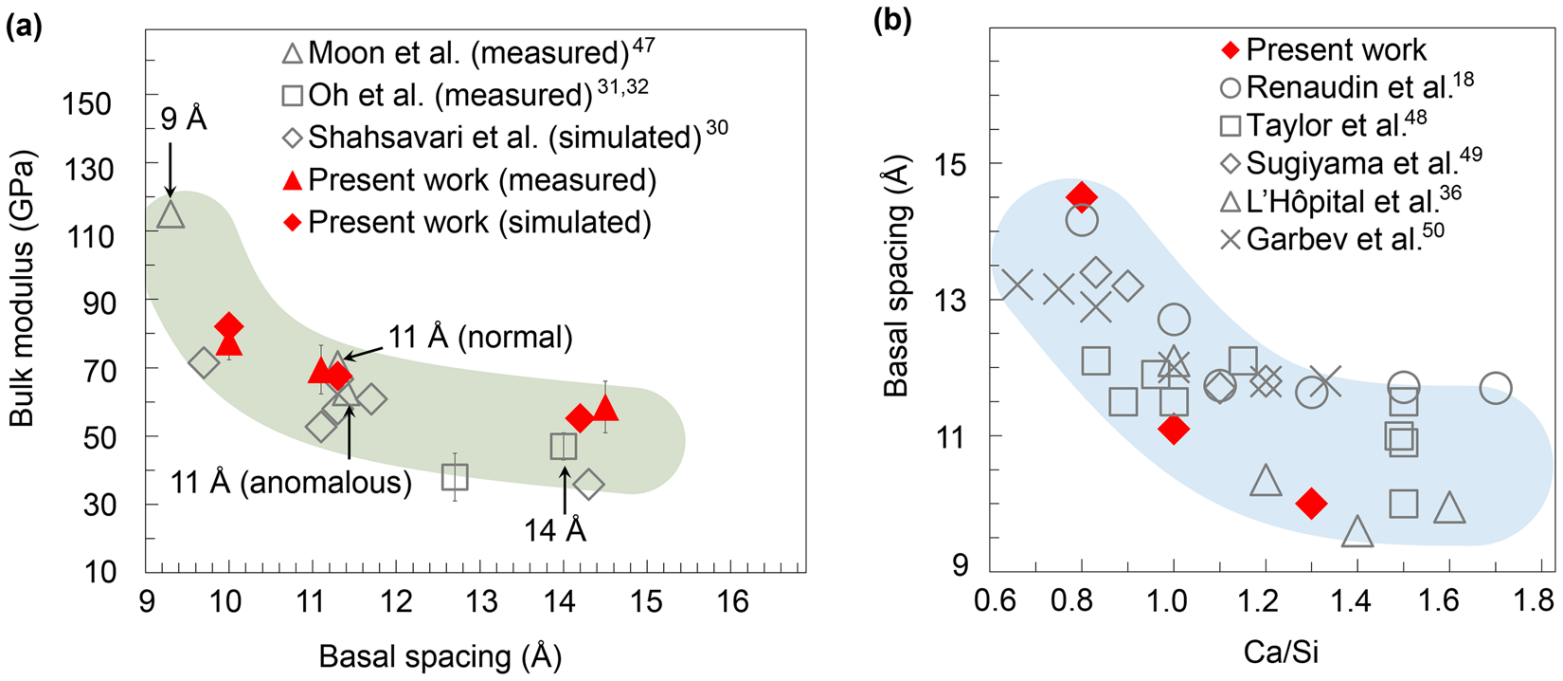
Composition-structure-mechanical property correlations in C-S-H. The green and blue shaded areas are eye-guides following the overall data trends. (**a**) The bulk modulus of C-S-H plotted as a function of the basal spacing^30–32, 47^. Experimentally measured results of crystalline tobermorite minerals are labelled with arrows. (**b**) C-S-H basal spacing plotted as a function of the Ca/Si^18, 36, 48–50^.

An inverse and non-linear correlation is readily observed when the measured bulk modulus of tobermorite^30, 31, 47^ is plotted as a function of the interlayer spacing (Fig. 4a). The atomistic simulation using the Hamid model of 11 Å tobermorite also yields proportional trends between the bulk modulus and the interlayer Ca content^30^, although the values calculated in those studies are systematically smaller and limited to a relatively narrow Ca/Si range. Published XRD measurements of C-S-H^18, 36, 48-50^ indicate that the interlayer spacing decreases with increasing Ca/Si ratio but plateaus at Ca/Si > 1.5 (Fig. 4b). These data suggest that the densification of the interlayer spacing may have an asymptotically minor influence at Ca/Si > 1.5, thus alternative processes might contribute to alteration of mechanical properties of C-S-H at higher Ca/Si. The poor crystallinity of C-S-H at Ca/Si > 1.5 poses a new challenge to observe this mechanism directly in HP-XRD experiments.

Overall, we consider data that can directly probe the atomic scale properties of C-S-H, such as those obtained here from HP-XRD measurements, as being essential to validate the accuracy, reliability and realism of atomistic C-S-H modeling results. The availability of such data will ultimately pave the way for constructing high fidelity C-S-H models that can assist us with understanding the fundamental physics of construction materials. Using such models, the intrinsic mechanical properties of C-S-H-based construction materials with changing chemical compositions and sub-nanometer configurations can be readily calculated. This scheme can be further used to investigate the evolution of mechanical properties of C-S-H after uptaking aluminum in the dreierketten chain and/or alkali in the interlayer, which is the fundamental input for designing the next generation of more durable and sustainable cementitious materials through tuning the chemistry and multiscale structure of the existing binding phases.

## Methods

### Synthesizing nanocrystalline C-S-H

C-S-H samples with bulk Ca/Si = ~0.80, ~1.0 and ~1.3 were synthesized by mixing stoichiometric amounts of CaO (obtained by burning CaCO_3_ (Merck Millipore) at 1000 °C for 12 h), SiO_2_ (Aerosil 200, Evonik), and Milli-Q water (Merck Millipore) in high density polyethylene bottles at a water to solid ratio = 45. The bottles were shaken at 100 rpm for 182 days. Solids were then collected by vacuum filtration in an N_2 (g)_-filled glove box using 0.45 μm nylon filters, washed with a 50% v/v ethanol/Milli-Q water (Merck Millipore) solution and then with ≥94 vol.% ethanol, freeze-dried for seven days, and then stored in N_2 (g)_-filled desiccators in the presence of a saturated CaCl_2_ solution (≈30% RH) and solid NaOH as a CO_2_ trap. In an order of increasing Ca/Si, samples were labeled 0.8CSH, 1.0CSH and 1.3CSH; the mean chain length (MCL) of these C-S-H materials were determined by ^29^Si magic angle spinning nuclear magnetic resonance (MAS NMR)^36^ to be 19, 5, and 2, respectively, which correspond to, on average, 15%, 50%, and 100% vacant bridging sites within the silicate chains of these C-S-H structures.

### High pressure XRD

The HP-XRD experiment was conducted at beamline 12.2.2 of the Advanced Light Source (ALS), Lawrence Berkeley National Laboratory (LBNL). Stainless steel gaskets and diamond anvils of culet diameter ~300 µm were used in an axial Merrell-Bassett cell. Hydrostatic pressure up to ~10 GPa, with step size 1–2 GPa, was generated by applying load on the diamond anvils. The experiment setting and data analysis algorithm are highly comparable to a previous study, and therefore is not repeated here for concision. Details are given in SI, including the determination of refinement uncertainty, and the application of the second order Birch–Murnaghan equation of state.

### Atomistic simulations

Atomistic models of 0.8CSH and 1.0CSH were proposed based on a 2 × 2 × 1 supercell of the 14 Å tobermorite structure^16^; the model of 1.3CSH was based on a 2 × 2 × 2 supercell of the 9 Å tobermorite structure^12^. Charge-neutral SiO_2_ groups of the supercells were first removed from the bridging silica tetrahedron to match the existing NMR data^36^. The Ca/Si ratios of the C-S-H models were then adjusted by modifying the amount of Ca in the interlayer space. We performed constant volume energy minimization to relax the atomic coordinates and simultaneously adjusted the amount of water molecules in the interlayer space, so that the ambient pressure lattice parameters match the experimental values obtained from refinement of our XRD data (see Supplementary Figure 2 in SI for details). In this work, we use transferrable CSH-FF potential to describe interatomic interactions; see SI for definition of the interatomic potential^43^. Once the ambient pressure C-S-H models were generated, we subsequently increased the external pressure up to 8 GPa with intervals of 0.1 GPa and relaxed both atomic positions and lattice vectors to locally minimize the enthalpy. We perform several loading-unloading sequences to ensure that our pressure-volume data is unaffected by the positions and local orientations of the nano-confined water molecules in the interlayer space. The pair distribution function, atomic distances and angles are calculated on the fully relaxed structures, (details in SI). Structural relaxation is performed by enthalpy minimization technique with a convergence norm tolerance of 10^−8^, while enforcing rational functional optimization below a norm of 10^−4^ to avoid unstable configurations. For this study, we use the GULP package^42^ along with a suite of pre- and post-processing tools to facilitate finite difference-based pressure-volumetric strain equation of state calculations.

## Electronic supplementary material

Cell parameters and fractional atom positions of 0.8CSH

Cell parameters and fractional atom positions of 1.0CSH

Cell parameters and fractional atom positions of 1.3CSH

supporting information
