# Supplementary material for: Densification of the interlayer spacing governs the nanomechanical properties of calcium-silicate-hydrate
Source: Sci Rep. 2017 Sep 8;7:10986. doi: 10.1038/s41598-017-11146-8 (PMC5591233; doi:10.1038/s41598-017-11146-8)
Supplement: Supplementary file 1 — Cell parameters and fractional atom positions of 0.8CSH [file 41598_2017_11146_MOESM1_ESM.pdf]

Table S6. Cell parameters and fractional atom positions of 0.8CSH.

a=13.466 Å, b=14.979 Å, c=28.455 Å, α=90.2°, β=87.3°, γ=124.3°			
Name of atoms	X	Y	Z
Ca	0.3685	0.2125	0.2836
Ca	0.36504	0.71231	0.27814
Ca	0.86752	0.21362	0.28566
Ca	0.86342	0.71005	0.28268
Ca	0.27831	0.46108	0.73064
Ca	0.27719	0.95887	0.72716
Ca	0.77587	0.45894	0.72452
Ca	0.77941	0.96263	0.72783
Ca	0.021	0.20982	0.79507
Ca	0.01531	0.70403	0.78908
Ca	0.51716	0.20404	0.79568
Ca	0.51681	0.70918	0.7911
Ca	0.12514	0.46659	0.21848
Ca	0.12138	0.96562	0.22026
Ca	0.62367	0.4651	0.21979
Ca	0.62248	0.96612	0.22327
Ca	0.13614	0.21858	0.21859
Ca	0.1338	0.71893	0.21809
Ca	0.63044	0.21319	0.2244
Ca	0.63394	0.72136	0.21961
Ca	0.03002	0.45821	0.79241
Ca	0.02912	0.95984	0.79242
Ca	0.52959	0.46064	0.79257
Ca	0.52584	0.95792	0.78727
Ca	0.28752	0.2133	0.73004
Ca	0.28429	0.71214	0.72681
Ca	0.7834	0.21111	0.73403
Ca	0.78454	0.71355	0.72776
Ca	0.3759	0.46501	0.2805
Ca	0.37182	0.96366	0.28627
Ca	0.88	0.46692	0.28439
Ca	0.8726	0.9656	0.28898
Si	0.38745	0.19739	0.17851
Si	0.39436	0.70532	0.17208
Si	0.88671	0.20142	0.18077
Si	0.88666	0.70223	0.1763
Si	0.27772	0.44098	0.83684
Si	0.26331	0.93159	0.83331
Si	0.77648	0.4397	0.83015
Si	0.76651	0.94044	0.83293
Si	0.0453	0.19822	0.68856
Si	0.04096	0.69414	0.68464
Si	0.53728	0.19406	0.68792

Si	0.54084	0.70032	0.68634
Si	0.12368	0.44704	0.32459
Si	0.11445	0.94109	0.32538
Si	0.62236	0.44153	0.32737
Si	0.60532	0.93703	0.32976
Si	0.47599	0.38561	0.10679
Si	0.46669	0.89508	0.10066
Si	0.98167	0.38968	0.10564
Si	0.95512	0.88729	0.10422
Si	0.32582	0.10278	0.90902
Si	0.34001	0.62473	0.90506
Si	0.82894	0.11385	0.90996
Si	0.13899	0.38933	0.61486
Si	0.12292	0.8836	0.61606
Si	0.61139	0.38096	0.61411
Si	0.60186	0.87122	0.60271
Si	0.17685	0.62189	0.39888
Si	0.62172	0.0846	0.41134
Si	0.6716	0.62047	0.40087
Si	0.38758	0.49154	0.17379
Si	0.38187	0.98659	0.18001
Si	0.89035	0.49208	0.17237
Si	0.88135	0.99063	0.18225
Si	0.27311	0.22728	0.83744
Si	0.26548	0.72546	0.83294
Si	0.76986	0.2292	0.84068
Si	0.76214	0.73013	0.83422
Si	0.04001	0.48188	0.6842
Si	0.03695	0.98495	0.68667
Si	0.53659	0.48767	0.68592
Si	0.54127	0.98712	0.68039
Si	0.1215	0.23734	0.32645
Si	0.11358	0.73186	0.32646
Si	0.62242	0.23179	0.3296
Si	0.60836	0.72777	0.325
Ob	0.39987	0.26417	0.12994
Ob	0.42847	0.77792	0.12219
Ob	0.90181	0.2745	0.13416
Ob	0.91119	0.76913	0.12568
Ob	0.26158	0.99315	0.88037
Ob	0.29559	0.5057	0.88725
Ob	0.76074	0.00384	0.87951
Oh	0.80817	0.52346	0.87847
Ob	0.0707	0.26962	0.63993
Ob	0.05888	0.76221	0.63581
Ob	0.54648	0.26239	0.63927

Ob	0.55382	0.76651	0.63786
Oh	0.11066	0.00328	0.37626
Ob	0.13196	0.51066	0.37345
Ob	0.57682	0.97763	0.38039
Ob	0.63342	0.50992	0.37478
Ob	0.38748	0.09326	0.15995
Ob	0.38082	0.59248	0.15515
Ob	0.88541	0.09648	0.16177
Ob	0.87819	0.59236	0.159
Ob	0.26668	0.33005	0.85425
Ob	0.25473	0.82325	0.85407
Ob	0.7653	0.33436	0.85256
Ob	0.75925	0.83255	0.85446
Ob	0.0379	0.09025	0.67022
Ob	0.03731	0.5867	0.66817
Ob	0.53884	0.09285	0.66633
Ob	0.53514	0.59237	0.6685
Ob	0.11892	0.34038	0.34269
Ob	0.11003	0.83564	0.34458
Ob	0.61185	0.33219	0.34725
Ob	0.6045	0.82987	0.34559
O	0.49771	0.27143	0.21324
O	0.4986	0.77571	0.20939
O	0.99662	0.27229	0.21574
O	0.99597	0.77424	0.21117
O	0.38517	0.006	0.79914
O	0.39226	0.51668	0.80029
O	0.88867	0.01564	0.79985
O	0.89191	0.51023	0.79327
O	0.14903	0.26694	0.72565
O	0.14953	0.76819	0.71941
O	0.64811	0.26518	0.72232
O	0.65167	0.77315	0.72016
O	0.2352	0.02283	0.29368
O	0.23829	0.52282	0.28765
O	0.72939	0.0254	0.30077
O	0.73666	0.51314	0.28895
O	0.26621	0.15713	0.21098
O	0.26978	0.66675	0.20378
O	0.76376	0.15834	0.21308
O	0.76528	0.66334	0.20891
O	0.16132	0.40527	0.80662
O	0.152	0.89365	0.79987
O	0.66144	0.40525	0.80089
O	0.65581	0.90154	0.79937
O	0.91835	0.15957	0.71739

O	0.91773	0.65678	0.71601
O	0.41693	0.15223	0.72084
O	0.41904	0.65867	0.7194
O	0.00691	0.40759	0.29395
O	0.00138	0.90364	0.29402
O	0.50629	0.40384	0.29524
O	0.49854	0.89991	0.29378
O	0.3308	0.09326	0.96413
Oh	0.31855	0.62477	0.96366
O	0.83584	0.09849	0.9643
Ow	0.86361	0.58389	0.9781
Oh	0.4758	0.37053	0.04826
O	0.45082	0.89442	0.04498
O	0.98252	0.37928	0.04925
O	0.94881	0.89328	0.04849
Ow	0.19313	0.21102	0.48999
Oh	0.15964	0.60331	0.45744
Oh	0.5288	0.06077	0.45824
O	0.64983	0.61893	0.45668
O	0.15609	0.38669	0.55856
Oh	0.12965	0.8857	0.55713
Oh	0.60559	0.37665	0.55579
O	0.56076	0.83583	0.55055
Oh	0.1256	0.46215	0.12566
Oh	0.10796	0.97651	0.11864
Oh	0.61724	0.45287	0.12587
Oh	0.61944	0.99139	0.11023
Oh	0.98108	0.18644	0.88491
Oh	0.03773	0.67681	0.90102
Oh	0.48077	0.17917	0.8852
Oh	0.48755	0.70861	0.88718
Oh	0.27882	0.46826	0.639
Oh	0.26384	0.96478	0.63512
Oh	0.75679	0.45213	0.62876
Oh	0.76064	0.95371	0.60124
Oh	0.36576	0.28302	0.41313
Oh	0.32882	0.71268	0.38762
Oh	0.75335	0.11957	0.4331
Oh	0.82205	0.71725	0.38491
Ow	0.16217	0.21709	0.13124
Ow	0.15374	0.73122	0.12909
Ow	0.67549	0.25105	0.12587
Ow	0.65284	0.73787	0.13508
Ow	0.032	0.46843	0.88273
Ow	0.01848	0.01424	0.8888
Ow	0.52497	0.45401	0.87971

Ow	0.52663	0.95763	0.88824
Ow	0.31528	0.22214	0.63924
Ow	0.30876	0.70958	0.64041
Ow	0.83621	0.22509	0.62161
Ow	0.81746	0.72382	0.63495
Ow	0.37707	0.45654	0.36799
Ow	0.35438	0.93816	0.37678
Ow	0.87893	0.48301	0.37215
Ow	0.85447	0.93473	0.38183
Ob	0.40621	0.43929	0.12492
Ob	0.39002	0.92793	0.13252
Ob	0.92201	0.45694	0.12019
Ob	0.88291	0.92633	0.13532
Ob	0.2748	0.1716	0.88875
Ob	0.2628	0.66056	0.88025
Ob	0.77429	0.18202	0.89381
Oh	0.74691	0.66083	0.88646
Ob	0.05992	0.43598	0.63311
Ob	0.04589	0.92948	0.6367
Ob	0.54962	0.43734	0.63617
Ob	0.55521	0.93904	0.62969
Oh	0.14186	0.1922	0.38021
Ob	0.11018	0.67249	0.37696
Ob	0.63316	0.17452	0.37665
Ob	0.59494	0.65951	0.37355
O	0.26634	0.40945	0.20702
O	0.26116	0.91154	0.21369
O	0.7634	0.39523	0.19819
O	0.76218	0.91738	0.21747
O	0.1581	0.14745	0.80604
O	0.15395	0.64994	0.79971
O	0.65557	0.1431	0.80943
O	0.65403	0.65527	0.80097
O	0.91561	0.39432	0.71388
O	0.91598	0.90332	0.71957
O	0.41722	0.4058	0.71945
O	0.42071	0.9041	0.71377
O	0.00226	0.15219	0.29994
O	0.00211	0.65698	0.29334
O	0.50639	0.15252	0.29769
O	0.50034	0.65626	0.29025
O	0.49379	0.02524	0.21368
O	0.4969	0.5278	0.20879
O	0.99403	0.02821	0.21496
O	0.99308	0.52551	0.20972
O	0.38983	0.26623	0.80261

O	0.386	0.77033	0.79942
O	0.88697	0.26536	0.80535
O	0.88475	0.7669	0.80459
O	0.14778	0.02032	0.72012
O	0.14625	0.51619	0.71991
O	0.65253	0.02474	0.71514
O	0.64899	0.52397	0.71907
O	0.23596	0.27113	0.29116
O	0.23275	0.76874	0.29308
O	0.73934	0.27678	0.29293
O	0.73187	0.76972	0.29399
Ow	0.38291	0.32344	0.95905
Ow	0.54985	0.85762	0.96962
Ow	0.85706	0.38314	0.95317
Ow	0.96533	0.82877	0.92126
Ow	0.48878	0.12834	0.06907
Ow	0.34397	0.49199	0.04314
Ow	0.93767	0.11043	0.06718
Ow	0.98226	0.66017	0.06257
Ow	0.98342	0.32053	0.43446
Ow	0.02381	0.80709	0.43725
Ow	0.58068	0.29925	0.44505
Ow	0.64793	0.84354	0.44019
Ow	0.16571	0.09798	0.57189
Ow	0.22517	0.66143	0.54971
Ow	0.6602	0.2051	0.57411
Ow	0.58437	0.60468	0.57011
Ow	0.0889	0.10815	0.98721
Ow	0.08733	0.60305	0.98107
Ow	0.60148	0.11183	0.97231
Ow	0.61116	0.60945	0.06274
Ow	0.27449	0.0254	0.43968
Ow	0.4292	0.62607	0.44349
Ow	0.85688	0.19246	0.5251
Ow	0.79977	0.63917	0.522
Cw	0.40715	0.00926	0.00609
Cw	0.9108	0.48974	0.02625
Cw	0.88518	0.99206	0.00666
Cw	0.05456	0.25954	0.49303
Cw	0.57072	0.70161	0.50687
Ow	0.26254	0.14074	0.0451
Ow	0.25462	0.69208	0.0452
Ow	0.77024	0.17434	0.03581
Ow	0.78552	0.72657	0.00139
Ow	0.008	0.43015	0.52169
Ow	0.00441	0.94934	0.49052

Ow	0.48155	0.42239	0.49003
Ow	0.4502	0.82848	0.47375
Ow	0.4774	0.55298	0.9565
Ow	0.36705	0.5879	0.54041
Ow	0.96869	0.55113	0.57683
Ow	0.43372	0.06394	0.55186
Ow	0.99808	0.13328	0.56525
Ow	0.70903	0.36863	0.04554
Ow	0.13052	0.30941	0.05534
Ow	0.71233	0.89146	0.05584
Ow	0.23441	0.92036	0.0567
Ow	0.22733	0.40943	0.44921
Ow	0.81553	0.51107	0.45845
Ow	0.25682	0.84082	0.46918
Ow	0.94305	0.08651	0.45541
Ow	0.62809	0.33276	0.94911
Ow	0.17606	0.29772	0.94505
Ow	0.75228	0.85996	0.95258
Ow	0.28683	0.86389	0.95505
Ow	0.34164	0.35486	0.56885
Ow	0.35843	0.86502	0.57465
Ow	0.02711	0.69555	0.50996
Ow	0.38663	0.17985	0.50148
Ow	0.76974	0.32454	0.49404
Ow	0.77444	0.82796	0.52723
Ow	0.09371	0.41474	0.96397
Ow	0.06303	0.82531	0.99711
Ow	0.4939	0.70488	0.04451
Ow	0.6333	0.50694	0.97409
H	0.89496	0.57471	0.88992
H	0.10947	0.07104	0.38242
H	0.30393	0.64776	0.99609
Hw	0.78335	0.57616	0.97541
H	0.44226	0.34909	0.01494
Hw	0.21177	0.17734	0.51641
H	0.18623	0.63123	0.49101
H	0.4821	0.04986	0.48959
H	0.09652	0.8211	0.53504
H	0.56855	0.38086	0.52571
H	0.21808	0.5082	0.12506
H	0.16491	0.05414	0.1052
H	0.70813	0.51015	0.12244
H	0.68846	0.06431	0.09559
H	0.05559	0.23209	0.90445
H	0.11905	0.73942	0.88625
H	0.54695	0.23886	0.90562

H	0.57658	0.74677	0.89352
H	0.3709	0.51227	0.63462
H	0.35047	0.01452	0.62111
H	0.83861	0.49362	0.60948
H	0.84496	0.01943	0.59086
H	0.43928	0.27917	0.41153
H	0.40998	0.78379	0.39251
H	0.81276	0.10185	0.44329
H	0.89917	0.74944	0.40327
Hw	0.15306	0.25407	0.10341
Hw	0.22343	0.80889	0.12156
Hw	0.70226	0.31409	0.14803
Hw	0.73521	0.80779	0.12831
Hw	0.1038	0.54713	0.88192
Hw	0.99748	0.06527	0.8773
Hw	0.59114	0.5269	0.89144
Hw	0.53702	0.0285	0.88123
Hw	0.333	0.28553	0.61814
Hw	0.32252	0.77267	0.62084
Hw	0.90249	0.30431	0.62625
Hw	0.87894	0.80459	0.63079
Hw	0.45991	0.52641	0.37013
Hw	0.29869	0.87586	0.39965
Hw	0.83571	0.48383	0.40251
Hw	0.78594	0.90775	0.4067
H	0.76336	0.60108	0.88976
H	0.22362	0.22711	0.39588
Hw	0.31016	0.32751	0.96368
Hw	0.52523	0.87925	0.9408
Hw	0.91411	0.42777	0.925
Hw	0.98537	0.89991	0.9066
Hw	0.4331	0.10721	0.09884
Hw	0.389	0.57419	0.04361
Hw	0.91702	0.1025	0.10288
Hw	7.51E-04	0.71374	0.03581
Hw	0.89805	0.28312	0.42421
Hw	0.06812	0.81766	0.40526
Hw	0.60744	0.31945	0.41022
Hw	0.59008	0.86289	0.45288
Hw	0.2311	0.15162	0.59343
Hw	0.27542	0.74371	0.55357
Hw	0.6094	0.21239	0.59966
Hw	0.59342	0.62958	0.60411
Hw	0.15183	0.11789	0.00944
Hw	0.1631	0.65614	0.99838
Hw	0.66364	0.13713	0.99752

Hw	0.55812	0.63501	0.05456
Hw	0.20614	0.01569	0.42107
Hw	0.38651	0.66023	0.43292
Hw	0.83807	0.19243	0.56019
Hw	0.76357	0.64966	0.5518
Hw	0.33759	0.19317	0.06222
Hw	0.22766	0.68128	0.07984
Hw	0.84854	0.24567	0.04269
Hw	0.85254	0.7882	0.02035
Hw	0.07054	0.42398	0.53802
Hw	0.07167	0.97505	0.51281
Hw	0.44698	0.37701	0.46104
Hw	0.48276	0.83961	0.50698
Hw	0.42119	0.52984	0.98579
Hw	0.3252	0.62726	0.54342
Hw	0.00764	0.56779	0.60871
Hw	0.40917	0.11136	0.53756
Hw	0.02093	0.1789	0.59486
Hw	0.67793	0.32246	0.07583
Hw	0.2148	0.37767	0.04775
Hw	0.63747	0.81453	0.05606
Hw	0.20203	0.94351	0.08375
Hw	0.2998	0.48469	0.45579
Hw	0.80127	0.55052	0.48418
Hw	0.25478	0.90771	0.4681
Hw	0.93925	0.064	0.42134
Hw	0.62649	0.39965	0.95368
Hw	0.14783	0.3477	0.95269
Hw	0.67488	0.85798	0.9575
Hw	0.29869	0.8554	0.91996
Hw	0.27503	0.36438	0.5613
Hw	0.43866	0.89864	0.59022
Hw	0.01915	0.64215	0.53495
Hw	0.33571	0.11873	0.47884
Hw	0.7208	0.28301	0.52423
Hw	0.77902	0.87578	0.55368
Hw	0.04224	0.39384	0.9951
Hw	0.02374	0.85804	0.01596
Hw	0.46103	0.72405	0.0734
Hw	0.6008	0.47966	0.00769
Hw	0.91992	0.63718	0.95193
Hw	0.26183	0.23571	0.46533
Hw	0.24675	0.2346	0.12732
Hw	0.09248	0.70796	0.10413
Hw	0.74511	0.24205	0.12542
Hw	0.64235	0.68315	0.11076

Hw	0.05328	0.43254	0.9069
Hw	0.10773	0.06377	0.8948
Hw	0.48254	0.40714	0.90922
Hw	0.60984	0.97167	0.88324
Hw	0.39437	0.228	0.63867
Hw	0.38635	0.71312	0.63693
Hw	0.86193	0.18427	0.63987
Hw	0.86626	0.69169	0.63253
Hw	0.3834	0.39728	0.3818
Hw	0.43542	0.94736	0.37809
Hw	0.96233	0.55205	0.37432
Hw	0.86819	0.87491	0.37965
Hw	0.3521	0.26238	0.9359
Hw	0.51907	0.8805	0.99792
Hw	0.82008	0.30715	0.94141
Hw	0.88395	0.76915	0.91002
Hw	0.56937	0.16961	0.08457
Hw	0.3699	0.47462	0.07326
Hw	0.01465	0.18669	0.06303
Hw	0.94024	0.67602	0.08893
Hw	0.03668	0.38123	0.41016
Hw	0.02594	0.74988	0.45545
Hw	0.65079	0.30313	0.45943
Hw	0.63018	0.83358	0.4051
Hw	0.11069	0.03426	0.59467
Hw	0.19764	0.63326	0.58345
Hw	0.72087	0.2035	0.59396
Hw	0.54403	0.52421	0.57572
Hw	0.12666	0.18293	0.97158
Hw	0.09156	0.63999	0.95015
Hw	0.65257	0.13928	0.9415
Hw	0.68154	0.64717	0.03778
Hw	0.32489	0.0156	0.41499
Hw	0.48483	0.63692	0.415
Hw	0.77975	0.16604	0.50905
Hw	0.74593	0.63599	0.49641
Hw	0.29216	0.12631	0.01355
Hw	0.32753	0.7714	0.04233
Hw	0.79793	0.1417	0.01057
Hw	0.76766	0.76281	0.97612
Hw	0.05344	0.48097	0.49332
Hw	0.01346	0.9012	0.46753
Hw	0.55087	0.49768	0.47709
Hw	0.38251	0.84028	0.47586
Hw	0.42356	0.52052	0.929
Hw	0.29641	0.50877	0.54481

Hw	0.98365	0.49825	0.56061
Hw	0.52239	0.11585	0.55901
Hw	0.05859	0.11221	0.56457
Hw	0.64203	0.32944	0.02275
Hw	0.07524	0.33397	0.04854
Hw	0.69528	0.92715	0.08261
Hw	0.16472	0.87961	0.03464
Hw	0.23601	0.40642	0.41355
Hw	0.79685	0.44183	0.47424
Hw	0.17559	0.78012	0.48377
Hw	0.9616	0.03754	0.47211
Hw	0.71604	0.36235	0.94154
Hw	0.22028	0.32306	0.91233
Hw	0.76293	0.85767	0.91673
Hw	0.20113	0.84385	0.9585
Hw	0.39396	0.3756	0.53822
Hw	0.36637	0.93263	0.56178
Hw	0.94095	0.67683	0.51252
Hw	0.46398	0.23246	0.48155
Hw	0.85624	0.35343	0.50135
Hw	0.85995	0.86843	0.51227
Hw	0.16191	0.49309	0.96813
Hw	0.03107	0.82188	0.96433
Hw	0.49732	0.74854	0.01628
Hw	0.58502	0.53746	0.96477
