# Supplementary material for: Densification of the interlayer spacing governs the nanomechanical properties of calcium-silicate-hydrate
Source: Sci Rep. 2017 Sep 8;7:10986. doi: 10.1038/s41598-017-11146-8 (PMC5591233; doi:10.1038/s41598-017-11146-8)
Supplement: Supplementary file 2 — Cell parameters and fractional atom positions of 1.0CSH [file 41598_2017_11146_MOESM2_ESM.pdf]

Table S7. Cell parameters and fractional atom positions of 1.0CSH.

a=13.449 Å, *b*=14.815 Å, *c*=22.595 Å, *α*=91.6°, *β*=86.5°, *γ*=125.1°			
Name of atoms	X	Y	Z
Ca	0.3685	0.2125	0.29452
Ca	0.35644	0.70357	0.29067
Ca	0.8685	0.2125	0.29452
Ca	0.85644	0.70357	0.29067
Ca	0.34761	0.3776	0.69942
Ca	0.3279	0.86039	0.69883
Ca	0.84761	0.3776	0.69942
Ca	0.8279	0.86039	0.69883
Ca	0.07996	0.12852	0.78069
Ca	0.07816	0.61772	0.78792
Ca	0.57996	0.12852	0.78069
Ca	0.57817	0.61772	0.78792
Ca	0.14098	0.45792	0.20252
Ca	0.14913	0.97134	0.19729
Ca	0.64098	0.45792	0.20252
Ca	0.64913	0.97134	0.19729
Ca	0.17642	0.22937	0.20559
Ca	0.17064	0.71889	0.19888
Ca	0.67642	0.22937	0.20559
Ca	0.67064	0.71889	0.19888
Ca	0.0947	0.38042	0.76993
Ca	0.08751	0.87502	0.77106
Ca	0.5947	0.38042	0.76993
Ca	0.58751	0.87502	0.77106
Ca	0.36548	0.13657	0.69944
Ca	0.37895	0.63422	0.69178
Ca	0.86548	0.13657	0.69944
Ca	0.87895	0.63422	0.69178
Ca	0.4018	0.49587	0.27725
Ca	0.39578	0.97605	0.28431
Ca	0.9018	0.49587	0.27725
Ca	0.89578	0.97605	0.28431
Si	0.44508	0.21911	0.16804
Si	0.42793	0.69043	0.16791
Si	0.94508	0.21911	0.16804
Si	0.92793	0.69043	0.16791
Si	0.33128	0.35816	0.83657
Si	0.33049	0.8573	0.83154
Si	0.83128	0.35816	0.83657
Si	0.83049	0.8573	0.83154
Si	0.14688	0.14655	0.65043
Si	0.13779	0.62532	0.66032
Si	0.64688	0.14655	0.65043

Si	0.63779	0.62532	0.66032
Si	0.12028	0.44384	0.34482
Si	0.12242	0.93858	0.32979
Si	0.62028	0.44384	0.34482
Si	0.62242	0.93858	0.32979
Si	0.41093	0.04427	0.9225
Si	0.42937	0.5551	0.9248
Si	0.91093	0.04427	0.9225
Si	0.92937	0.5551	0.9248
Si	0.18962	0.1121	0.43048
Si	0.20448	0.63495	0.43781
Si	0.68962	0.1121	0.43048
Si	0.70448	0.63495	0.43781
Si	0.40783	0.48299	0.14589
Si	0.4163	0.99492	0.15293
Si	0.90783	0.48299	0.14589
Si	0.9163	0.99492	0.15293
Si	0.33049	0.1471	0.83824
Si	0.33851	0.64593	0.83931
Si	0.83049	0.1471	0.83824
Si	0.83851	0.64593	0.83931
Si	0.11598	0.40606	0.6361
Si	0.10651	0.90928	0.63849
Si	0.61598	0.40606	0.6361
Si	0.60651	0.90928	0.63849
Si	0.12265	0.23436	0.35754
Si	0.11089	0.72155	0.35681
Si	0.62265	0.23436	0.35754
Si	0.61089	0.72155	0.35681
O	0.52675	0.32841	0.12897
O	0.51816	0.81304	0.1424
O	0.02675	0.32841	0.12897
O	0.01816	0.81304	0.1424
Ob	0.33913	0.92455	0.89265
Ob	0.34798	0.43205	0.89703
Ob	0.83913	0.92455	0.89265
Ob	0.84798	0.43205	0.89703
O	0.22784	0.24118	0.60157
O	0.1968	0.73994	0.62637
O	0.72784	0.24118	0.60157
O	0.6968	0.73994	0.62637
Ob	0.11538	0.00883	0.38857
Ob	0.13502	0.51806	0.40664
Ob	0.61538	0.00883	0.38857
Ob	0.63502	0.51806	0.40664
Ob	0.41941	0.10617	0.1335

Ob	0.41313	0.59539	0.11854
Ob	0.91941	0.10617	0.1335
Ob	0.91313	0.59539	0.11854
Ob	0.30875	0.24263	0.86087
Ob	0.32803	0.75122	0.85622
Ob	0.80875	0.24263	0.86087
Ob	0.82803	0.75122	0.85622
Ob	0.11292	0.02478	0.62006
Ob	0.13208	0.52937	0.61532
Ob	0.61292	0.02478	0.62006
Ob	0.63208	0.52937	0.61532
Ob	0.12173	0.34283	0.37337
Ob	0.12182	0.83945	0.36183
Ob	0.62173	0.34283	0.37337
Ob	0.62182	0.83945	0.36183
O	0.523	0.24896	0.22775
O	0.48243	0.67892	0.22776
O	0.023	0.24896	0.22775
O	0.98243	0.67892	0.22776
O	0.44741	0.93424	0.78496
O	0.45122	0.42516	0.79116
O	0.94741	0.93424	0.78496
O	0.95122	0.42516	0.79116
O	0.23202	0.18299	0.70657
O	0.22536	0.65558	0.71421
O	0.73202	0.18299	0.70657
O	0.72536	0.65558	0.71421
O	0.24624	0.0201	0.29019
O	0.23103	0.5131	0.29625
O	0.74624	0.0201	0.29019
O	0.73103	0.5131	0.29625
O	0.31368	0.18698	0.19481
O	0.29559	0.6599	0.19057
O	0.81368	0.18698	0.19481
O	0.79559	0.6599	0.19057
O	0.21933	0.32607	0.79561
O	0.21351	0.81534	0.79214
O	0.71933	0.32607	0.79561
O	0.71351	0.81534	0.79214
O	0.0212	0.12093	0.67933
O	0.00318	0.57375	0.68984
O	0.5212	0.12093	0.67933
O	0.50318	0.57375	0.68984
O	0.99543	0.40345	0.31357
O	0.01118	0.89177	0.28681
O	0.49543	0.40345	0.31357

O	0.51118	0.89177	0.28681
Oh	0.41438	0.03519	0.99468
Oh	0.43655	0.55529	0.99699
Oh	0.91438	0.03519	0.99468
Oh	0.93655	0.55529	0.99699
Oh	0.16653	0.07235	0.50158
Oh	0.20426	0.63504	0.51241
Oh	0.66653	0.07235	0.50158
Oh	0.70426	0.63504	0.51241
Oh	0.22909	0.56786	0.05199
Oh	0.22258	0.04579	0.07892
Oh	0.72909	0.56786	0.05199
Oh	0.72258	0.04579	0.07892
Oh	0.06093	0.11865	0.89541
Oh	0.07741	0.62189	0.89646
Oh	0.56093	0.11865	0.89541
Oh	0.57741	0.62189	0.89646
Ow	0.43353	0.48496	0.55734
Ow	0.40701	0.9963	0.56541
Ow	0.93353	0.48496	0.55734
Ow	0.90701	0.9963	0.56541
Ow	0.3468	0.17142	0.41171
Ow	0.36041	0.70179	0.41299
Ow	0.8468	0.17142	0.41171
Ow	0.86041	0.70179	0.41299
Ow	0.18701	0.21696	0.09166
Ow	0.20264	0.73701	0.08793
Ow	0.68701	0.21696	0.09166
Ow	0.70264	0.73701	0.08793
Ow	0.1035	0.43632	0.93031
Ow	0.11704	0.9904	0.95666
Ow	0.6035	0.43632	0.93031
Ow	0.61704	0.9904	0.95666
Ow	0.38136	0.42565	0.4131
Ow	0.37619	0.91125	0.38567
Ow	0.88136	0.42565	0.4131
Ow	0.87619	0.91125	0.38567
O	0.39727	0.40891	0.09204
O	0.41981	0.93114	0.09616
O	0.89727	0.40891	0.09204
O	0.91981	0.93114	0.09616
Ob	0.35037	0.10461	0.90223
Ob	0.3785	0.62164	0.90202
Ob	0.85037	0.10461	0.90223
Ob	0.8785	0.62164	0.90202
O	0.10511	0.34557	0.57476

O	0.09749	0.8477	0.57747
O	0.60511	0.34557	0.57476
O	0.59749	0.8477	0.57747
Ob	0.16286	0.20322	0.42521
Ob	0.15701	0.70808	0.42317
Ob	0.66286	0.20322	0.42521
Ob	0.65701	0.70808	0.42317
O	0.28936	0.41939	0.19228
O	0.29068	0.91306	0.19432
O	0.78936	0.41939	0.19228
O	0.79068	0.91306	0.19432
O	0.21531	0.0514	0.80388
O	0.20959	0.54218	0.81971
O	0.71531	0.0514	0.80388
O	0.70959	0.54218	0.81971
O	0.99375	0.33609	0.67874
O	0.98384	0.82954	0.68047
O	0.49375	0.33609	0.67874
O	0.48384	0.82954	0.68047
O	0.98707	0.13428	0.34229
O	0.96868	0.62143	0.35301
O	0.48707	0.13428	0.34229
O	0.46868	0.62143	0.35301
O	0.52687	0.0376	0.19674
O	0.5223	0.52324	0.1863
O	0.02687	0.0376	0.19674
O	0.0223	0.52324	0.1863
O	0.4491	0.19246	0.79291
O	0.44488	0.68086	0.78772
O	0.9491	0.19246	0.79291
O	0.94488	0.68086	0.78772
O	0.21908	0.94413	0.67942
O	0.22668	0.44085	0.67733
O	0.71908	0.94413	0.67942
O	0.72668	0.44085	0.67733
O	0.22067	0.25057	0.3068
O	0.19351	0.72031	0.30239
O	0.72067	0.25057	0.3068
O	0.69351	0.72031	0.30239
Ow	0.38024	0.16034	0.58454
Ow	0.40347	0.64404	0.5808
Ow	0.88024	0.16034	0.58454
Ow	0.90347	0.64404	0.5808
Ow	0.15931	0.32364	0.00492
Ow	0.14821	0.81671	0.99845
Ow	0.65931	0.32364	0.00492

Ow	0.64821	0.81671	0.99845
Ow	0.45695	0.33143	0.48978
Ow	0.44701	0.85255	0.50526
Ow	0.95695	0.33143	0.48978
Ow	0.94701	0.85255	0.50526
Cw	0.2723	0.33539	0.50988
Cw	0.24631	0.82375	0.53275
Cw	0.7723	0.33539	0.50988
Cw	0.74631	0.82375	0.53275
Cw	0.53915	0.3936	0.03273
Cw	0.54006	0.88193	0.04373
Cw	0.03915	0.3936	0.03273
Cw	0.04006	0.88193	0.04373
Ow	0.3712	0.22888	0.99922
Ow	0.37384	0.76756	0.9854
Ow	0.8712	0.22888	0.99922
Ow	0.87384	0.76756	0.9854
Ow	0.2467	0.47236	0.48695
Ow	0.24138	0.9364	0.46458
Ow	0.7467	0.47236	0.48695
Ow	0.74138	0.9364	0.46458
H	0.41647	0.00816	0.03613
H	0.43212	0.56038	0.0427
H	0.91647	0.00816	0.03613
H	0.93212	0.56038	0.0427
H	0.15671	0.06098	0.54757
H	0.1784	0.59246	0.553
H	0.65671	0.06098	0.54757
H	0.6784	0.59246	0.553
H	0.29745	0.57339	0.07411
H	0.30582	0.06974	0.09458
H	0.79745	0.57339	0.07411
H	0.80582	0.06974	0.09458
Hw	0.42497	0.54774	0.56916
Hw	0.39625	0.05908	0.57324
Hw	0.92497	0.54774	0.56916
Hw	0.89625	0.05908	0.57324
Hw	0.2734	0.29098	0.08988
Hw	0.28818	0.8106	0.089
Hw	0.7734	0.29098	0.08988
Hw	0.78818	0.8106	0.089
Hw	0.086	0.0278	0.93493
Hw	0.12479	0.48119	0.89224
Hw	0.586	0.0278	0.93493
Hw	0.62479	0.48119	0.89224
Hw	0.34254	0.94119	0.4155

Hw	0.4073	0.50327	0.40218
Hw	0.84254	0.94119	0.4155
Hw	0.9073	0.50327	0.40218
Hw	0.33138	0.19337	0.58576
Hw	0.35231	0.63699	0.54762
Hw	0.83138	0.19337	0.58576
Hw	0.85231	0.63699	0.54762
Hw	0.15429	0.27554	0.03843
Hw	0.16503	0.77682	0.02835
Hw	0.65429	0.27554	0.03843
Hw	0.66503	0.77682	0.02835
Hw	0.50916	0.33433	0.52222
Hw	0.49797	0.8415	0.53269
Hw	0.00916	0.33433	0.52222
Hw	0.99797	0.8415	0.53269
Hw	0.3164	0.15949	0.02457
Hw	0.31912	0.68594	0.99699
Hw	0.8164	0.15949	0.02457
Hw	0.81912	0.68594	0.99699
Hw	0.21215	0.97916	0.48604
Hw	0.18366	0.43743	0.52249
Hw	0.71215	0.97916	0.48604
Hw	0.68366	0.43743	0.52249
Hw	0.50525	0.49929	0.5795
Hw	0.47908	0.01741	0.58939
Hw	0.00525	0.49929	0.5795
Hw	0.97908	0.01741	0.58939
Hw	0.19946	0.15545	0.08776
Hw	0.21457	0.67668	0.07596
Hw	0.69946	0.15545	0.08776
Hw	0.71457	0.67668	0.07596
Hw	0.15645	0.40819	0.92447
Hw	0.2008	0.02263	0.93609
Hw	0.65645	0.40819	0.92447
Hw	0.7008	0.02263	0.93609
Hw	0.41762	0.4072	0.37771
Hw	0.468	0.95454	0.39027
Hw	0.91762	0.4072	0.37771
Hw	0.968	0.95454	0.39027
Hw	0.47011	0.22792	0.58201
Hw	0.48074	0.7247	0.57944
Hw	0.97011	0.22792	0.58201
Hw	0.98074	0.7247	0.57944
Hw	0.23882	0.34975	0.98219
Hw	0.20776	0.83527	0.96328
Hw	0.73882	0.34975	0.98219

Hw	0.70776	0.83527	0.96328
Hw	0.51286	0.40015	0.46399
Hw	0.50294	0.93552	0.49913
Hw	0.01286	0.40015	0.46399
Hw	0.00294	0.93552	0.49913
Hw	0.36479	0.19881	0.95726
Hw	0.37378	0.76549	0.94025
Hw	0.86479	0.19881	0.95726
Hw	0.87378	0.76549	0.94025
Hw	0.18837	0.89983	0.42864
Hw	0.20137	0.46144	0.4499
Hw	0.68837	0.89983	0.42864
Hw	0.70137	0.46144	0.4499
H	0.15494	0.17452	0.8924
H	0.17109	0.66797	0.89576
H	0.65494	0.17452	0.8924
H	0.67109	0.66797	0.89576
Hw	0.40885	0.23398	0.43857
Hw	0.42818	0.77297	0.43199
Hw	0.90885	0.23398	0.43857
Hw	0.92818	0.77297	0.43199
Hw	0.39841	0.1475	0.38959
Hw	0.40397	0.66699	0.39453
Hw	0.89841	0.1475	0.38959
