# Supplementary material for: Densification of the interlayer spacing governs the nanomechanical properties of calcium-silicate-hydrate
Source: Sci Rep. 2017 Sep 8;7:10986. doi: 10.1038/s41598-017-11146-8 (PMC5591233; doi:10.1038/s41598-017-11146-8)
Supplement: Supplementary file 3 — Cell parameters and fractional atom positions of 1.3CSH [file 41598_2017_11146_MOESM3_ESM.pdf]

Table S8. Cell parameters and fractional atom positions of 1.3CSH.

a=22.348 Å, *b*=14.677 Å, *c*=19.983 Å, *α*=102.4°, *β*=94.6°, *γ*=90.6°			
Name of atoms	X	Y	Z
Si	0.19295	0.1079	0.1117
Si	0.19668	0.09501	0.61876
Si	0.18987	0.60623	0.11013
Si	0.19105	0.59211	0.61372
Si	0.69235	0.10363	0.1075
Si	0.69318	0.09401	0.61767
Si	0.6906	0.60589	0.1119
Si	0.69097	0.59075	0.6108
Si	0.30552	0.3679	0.40789
Si	0.3063	0.38452	0.90532
Si	0.30057	0.87	0.40659
Si	0.29968	0.88334	0.90359
Si	0.80537	0.3695	0.40686
Si	0.805	0.38299	0.9071
Si	0.80251	0.87218	0.40734
Si	0.80186	0.88096	0.9017
Si	0.44181	0.35645	0.11136
Si	0.44885	0.34782	0.618
Si	0.44123	0.85571	0.10826
Si	0.44075	0.84201	0.6153
Si	0.94223	0.35476	0.11078
Si	0.94636	0.34052	0.61512
Si	0.9411	0.85229	0.10984
Si	0.94038	0.84208	0.61112
Si	0.05402	0.11854	0.40776
Si	0.0484	0.12937	0.89876
Si	0.05464	0.61628	0.40328
Si	0.04895	0.62787	0.89909
Si	0.55306	0.12036	0.40702
Si	0.55247	0.13338	0.9036
Si	0.55425	0.61968	0.39931
Si	0.54971	0.63286	0.90645
Si	0.19519	0.3142	0.11277
Si	0.19828	0.30273	0.61164
Si	0.19158	0.81917	0.11913
Si	0.19331	0.79958	0.61304
Si	0.69504	0.3138	0.11405
Si	0.69886	0.3004	0.61221
Si	0.69177	0.81786	0.11942
Si	0.69486	0.8001	0.6112
Si	0.30479	0.15588	0.39924
Si	0.30163	0.17425	0.89953
Si	0.29992	0.6588	0.39984

Si	0.29557	0.67414	0.90253
Si	0.80356	0.15638	0.39614
Si	0.80095	0.17248	0.90155
Si	0.80061	0.66032	0.39772
Si	0.79739	0.67157	0.90042
Si	0.44351	0.06788	0.11832
Si	0.44412	0.05264	0.61774
Si	0.44197	0.56927	0.12124
Si	0.4497	0.55277	0.6077
Si	0.94251	0.06542	0.11795
Si	0.94645	0.05289	0.61897
Si	0.94618	0.56541	0.11545
Si	0.94485	0.54933	0.60872
Si	0.05312	0.40268	0.39437
Si	0.05179	0.42001	0.90002
Si	0.0518	0.90997	0.40298
Si	0.05324	0.92442	0.90655
Si	0.55402	0.40452	0.39324
Si	0.54882	0.42514	0.90229
Si	0.55017	0.91143	0.40461
Si	0.54851	0.92284	0.90104
Oh	0.20137	0.06555	0.18589
Oh	0.2058	0.06279	0.69751
Oh	0.19051	0.56349	0.18472
Oh	0.18486	0.55309	0.69055
Oh	0.70053	0.0628	0.18437
Oh	0.68621	0.05591	0.69433
Oh	0.6921	0.56224	0.18623
Oh	0.68612	0.55072	0.68671
Oh	0.30056	0.40841	0.32977
Oh	0.3116	0.42248	0.82465
Oh	0.28642	0.90791	0.33014
Oh	0.28829	0.91856	0.82392
Oh	0.80433	0.41543	0.33378
Oh	0.80902	0.41919	0.82564
Oh	0.79308	0.91388	0.33106
Oh	0.79231	0.9166	0.82286
Oh	0.44992	0.31558	0.18772
Oh	0.46262	0.31417	0.69734
Oh	0.45003	0.81341	0.18468
Oh	0.43313	0.80486	0.69179
Oh	0.95031	0.31374	0.18599
Oh	0.95786	0.31338	0.69709
Oh	0.94331	0.80803	0.18347
Oh	0.93669	0.80311	0.69066
Oh	0.04536	0.15988	0.33179

Oh	0.0364	0.16244	0.82049
Oh	0.05195	0.65404	0.32519
Oh	0.03807	0.65897	0.8168
Oh	0.54664	0.16391	0.33376
Oh	0.54231	0.1652	0.82194
Oh	0.55285	0.65713	0.32205
Oh	0.5449	0.67359	0.82932
H	0.21282	0.00139	0.19616
H	0.18831	0.06018	0.74376
H	0.20165	0.49812	0.19345
H	0.14379	0.52476	0.69861
H	0.71049	0.99739	0.19382
H	0.64464	0.02519	0.69823
H	0.70294	0.4964	0.19424
H	0.64555	0.52401	0.69779
H	0.29086	0.47496	0.32211
H	0.35142	0.45492	0.81662
H	0.28624	0.97563	0.32215
H	0.28604	0.98903	0.82249
H	0.79323	0.47929	0.32225
H	0.85025	0.44717	0.81758
H	0.78225	0.97865	0.32114
H	0.78836	0.9871	0.82128
H	0.45707	0.24786	0.19471
H	0.46409	0.24329	0.69608
H	0.46064	0.74748	0.19276
H	0.39277	0.77482	0.7006
H	0.9621	0.24959	0.19585
H	0.95815	0.24269	0.69814
H	0.95275	0.74498	0.19745
H	0.89477	0.77545	0.69726
H	0.03714	0.2257	0.3217
H	0.03815	0.23334	0.82022
H	0.04703	0.72302	0.3203
H	0.0315	0.72941	0.81775
H	0.53172	0.22367	0.31902
H	0.53909	0.23656	0.82337
H	0.54699	0.72406	0.31323
H	0.5359	0.74124	0.82416
Oh	0.2179	0.39539	0.1956
Oh	0.21715	0.38575	0.68932
Oh	0.19787	0.89199	0.20381
Oh	0.20004	0.88253	0.69202
Oh	0.7165	0.39179	0.19641
Oh	0.71796	0.38372	0.68952
Oh	0.69988	0.89213	0.20544

Oh	0.70736	0.87904	0.6905
Oh	0.30589	0.07896	0.31782
Oh	0.29923	0.09433	0.81904
Oh	0.27975	0.57955	0.31927
Oh	0.2771	0.59191	0.82502
Oh	0.79567	0.08436	0.30963
Oh	0.79903	0.09328	0.82105
Oh	0.78099	0.58359	0.31572
Oh	0.77866	0.58976	0.82327
Oh	0.4504	0.14308	0.19945
Oh	0.44742	0.1331	0.70071
Oh	0.44773	0.64326	0.20409
Oh	0.47174	0.63738	0.68365
Oh	0.95091	0.13875	0.20357
Oh	0.95475	0.13386	0.69788
Oh	0.97119	0.64016	0.19681
Oh	0.95681	0.62884	0.69001
Oh	0.04661	0.32928	0.31236
Oh	0.05139	0.33757	0.81997
Oh	0.03559	0.82779	0.32355
Oh	0.0524	0.83766	0.82708
Oh	0.54605	0.33423	0.30727
Oh	0.54193	0.34226	0.82301
Oh	0.52859	0.83213	0.32461
Oh	0.53054	0.84665	0.81714
H	0.26186	0.42092	0.19746
H	0.20827	0.45642	0.69381
H	0.18516	0.93329	0.24927
H	0.2107	0.95312	0.69712
H	0.76152	0.41255	0.20072
H	0.70944	0.45432	0.69305
H	0.6841	0.93217	0.24963
H	0.7098	0.95148	0.69951
H	0.31028	0.0483	0.26668
H	0.29173	0.07541	0.76624
H	0.23457	0.55937	0.31292
H	0.28663	0.52192	0.82195
H	0.80953	0.04375	0.26454
H	0.8081	0.10388	0.77324
H	0.73603	0.56241	0.30921
H	0.78595	0.51909	0.82067
H	0.43767	0.17335	0.24775
H	0.43687	0.17158	0.74804
H	0.43221	0.67896	0.24929
H	0.46399	0.70864	0.69253
H	0.93357	0.18061	0.24598

H	0.97297	0.14522	0.74831
H	0.01398	0.67058	0.20034
H	0.95929	0.70032	0.69235
H	0.05625	0.29591	0.26333
H	0.05511	0.31384	0.76794
H	0.99119	0.80347	0.31406
H	0.09417	0.80761	0.81957
H	0.55953	0.29082	0.26307
H	0.52355	0.32826	0.77269
H	0.48586	0.80145	0.31815
H	0.54698	0.81759	0.77043
O	0.24745	0.32004	0.0649
O	0.25005	0.30169	0.56231
O	0.24554	0.83196	0.0749
O	0.24881	0.8049	0.56998
O	0.74688	0.32062	0.0656
O	0.7507	0.29786	0.56266
O	0.7462	0.82936	0.07556
O	0.74764	0.80341	0.56429
O	0.24934	0.14272	0.44119
O	0.2459	0.16662	0.94208
O	0.24839	0.65163	0.44871
O	0.24476	0.67268	0.95391
O	0.74965	0.14268	0.44035
O	0.74675	0.16317	0.94618
O	0.74853	0.65121	0.4455
O	0.74734	0.67324	0.95255
O	0.49738	0.07659	0.0724
O	0.49915	0.06168	0.5751
O	0.49564	0.57771	0.07519
O	0.49993	0.55043	0.55533
O	0.9969	0.07648	0.07393
O	1.467E-4	0.05886	0.57286
O	0.99796	0.56873	0.06609
O	0.99816	0.55355	0.56123
O	0.9994	0.39209	0.44013
O	0.99747	0.41047	0.94486
O	0.99871	0.90465	0.45051
O	0.99778	0.91797	0.94927
O	0.49981	0.39024	0.43682
O	0.49577	0.41836	0.94979
O	0.49775	0.90684	0.45325
O	0.49478	0.91956	0.94744
O	0.24687	0.07969	0.06553
O	0.24975	0.0642	0.57133
O	0.24568	0.57347	0.06791

O	0.2479	0.56063	0.57274
O	0.74714	0.07345	0.06388
O	0.74819	0.06156	0.57359
O	0.74639	0.57525	0.0691
O	0.74719	0.55849	0.56914
O	0.25042	0.40095	0.45033
O	0.24959	0.41731	0.94472
O	0.24751	0.89768	0.4538
O	0.24536	0.91702	0.94878
O	0.75005	0.39999	0.45015
O	0.74902	0.42033	0.94726
O	0.74794	0.90142	0.45191
O	0.74694	0.9094	0.94672
O	0.99666	0.32558	0.06533
O	0.00127	0.30928	0.56955
O	0.99646	0.82353	0.06604
O	0.99609	0.80813	0.56975
O	0.4972	0.32699	0.06782
O	0.50226	0.31446	0.5702
O	0.49604	0.82706	0.06425
O	0.49678	0.80753	0.57363
O	0.49907	0.14814	0.45337
O	0.49746	0.164	0.94785
O	0.49819	0.65423	0.44131
O	0.49503	0.66512	0.95093
O	0.9994	0.14699	0.4524
O	0.99591	0.15897	0.94714
O	0.99861	0.64992	0.44466
O	0.99602	0.66222	0.94591
O	0.13342	0.32585	0.07172
O	0.1354	0.31789	0.57374
O	0.13054	0.82622	0.0744
O	0.13288	0.8076	0.56838
O	0.63285	0.32887	0.0749
O	0.63646	0.31572	0.5735
O	0.63119	0.82874	0.07553
O	0.63224	0.81085	0.57088
O	0.36493	0.15142	0.4468
O	0.36167	0.16559	0.94572
O	0.36267	0.64624	0.43956
O	0.35862	0.65639	0.94022
O	0.86443	0.14587	0.44012
O	0.86205	0.1624	0.94579
O	0.86288	0.64603	0.43756
O	0.86083	0.65212	0.9367
O	0.38247	0.07549	0.07281

O	0.38343	0.06101	0.57372
O	0.38094	0.57981	0.07669
O	0.38661	0.56729	0.56999
O	0.88181	0.07359	0.07348
O	0.88481	0.06297	0.57528
O	0.88435	0.58333	0.07647
O	0.88349	0.56366	0.5675
O	0.1144	0.39423	0.43935
O	0.11267	0.41437	0.94562
O	0.11331	0.90094	0.44588
O	0.11364	0.91882	0.95154
O	0.61491	0.39441	0.43727
O	0.61042	0.41589	0.94571
O	0.61197	0.89955	0.44568
O	0.60934	0.90683	0.94276
O	0.13117	0.079	0.06638
O	0.13498	0.05965	0.57406
O	0.12987	0.5783	0.06147
O	0.13183	0.55847	0.56469
O	0.63121	0.07252	0.06268
O	0.63289	0.06195	0.56914
O	0.63013	0.57626	0.06464
O	0.63114	0.55946	0.56204
O	0.36656	0.39818	0.45318
O	0.36548	0.41707	0.95272
O	0.36272	0.90313	0.44882
O	0.36059	0.91765	0.94771
O	0.86626	0.39608	0.45439
O	0.86458	0.41656	0.95392
O	0.86367	0.9046	0.45149
O	0.86236	0.9159	0.9468
O	0.38129	0.32417	0.06599
O	0.38729	0.30993	0.57486
O	0.38003	0.82372	0.06363
O	0.38134	0.81178	0.56543
O	0.88098	0.32223	0.06615
O	0.88578	0.2995	0.57177
O	0.88051	0.82272	0.06194
O	0.88066	0.80954	0.56404
O	0.11519	0.14954	0.45301
O	0.11029	0.16786	0.94259
O	0.11478	0.64787	0.44954
O	0.11105	0.66771	0.93874
O	0.61476	0.15118	0.45251
O	0.61255	0.17055	0.94846
O	0.61412	0.65142	0.44717

O	0.6112	0.66676	0.94969
Ob	0.19367	0.22156	0.14697
Ob	0.1957	0.21067	0.64744
Ob	0.19368	0.71923	0.14578
Ob	0.19434	0.7056	0.64732
Ob	0.6936	0.21681	0.14388
Ob	0.69709	0.20852	0.64883
Ob	0.6931	0.71897	0.14723
Ob	0.69429	0.70416	0.64359
Ob	0.30232	0.25517	0.3713
Ob	0.30268	0.27196	0.8696
Ob	0.30082	0.75565	0.37057
Ob	0.29467	0.7699	0.87266
Ob	0.80185	0.25666	0.36989
Ob	0.7976	0.2709	0.87407
Ob	0.80139	0.75895	0.37036
Ob	0.80114	0.7667	0.86874
Ob	0.44248	0.46923	0.1474
Ob	0.45158	0.46184	0.64426
Ob	0.44363	0.96781	0.14579
Ob	0.44585	0.95638	0.64868
Ob	0.94245	0.46881	0.14524
Ob	0.94734	0.45445	0.63978
Ob	0.94348	0.96563	0.14586
Ob	0.94606	0.95466	0.64781
Ob	0.05229	0.0054	0.37077
Ob	0.05115	0.01469	0.86958
Ob	0.05183	0.50326	0.36896
Ob	0.04449	0.51483	0.86912
Ob	0.55219	0.00633	0.37177
Ob	0.55223	0.01968	0.87202
Ob	0.55166	0.50576	0.3683
Ob	0.54313	0.52063	0.87164
Oh	0.07905	0.48908	0.18942
Oh	0.08554	0.45027	0.70386
Oh	0.07853	0.01334	0.19248
Oh	0.08014	0.95293	0.69425
Oh	0.57839	0.4966	0.19277
Oh	0.58667	0.45594	0.70789
Oh	0.57902	0.0096	0.19268
Oh	0.58206	0.95887	0.6982
Oh	0.41851	0.97485	0.32105
Oh	0.41463	0.02072	0.82497
Oh	0.41673	0.46599	0.32315
Oh	0.40825	0.53013	0.81095
Oh	0.91705	0.96966	0.32104

Oh	0.91767	0.01156	0.81668
Oh	0.91883	0.46892	0.3222
Oh	0.90705	0.52536	0.81385
Oh	0.32834	0.24911	0.19067
Oh	0.32785	0.20786	0.69484
Oh	0.3271	0.76427	0.19152
Oh	0.33225	0.71139	0.70937
Oh	0.82817	0.24447	0.19035
Oh	0.83067	0.18655	0.72035
Oh	0.82659	0.76282	0.19333
Oh	0.83057	0.71048	0.69748
Oh	0.16883	0.21788	0.32359
Oh	0.16884	0.25792	0.82428
Oh	0.16722	0.73153	0.32501
Oh	0.15875	0.74567	0.82257
Oh	0.66749	0.21039	0.32242
Oh	0.66394	0.27767	0.82332
Oh	0.66753	0.73465	0.32643
Oh	0.66679	0.7311	0.82234
H	0.11866	0.52609	0.19018
H	0.09399	0.38641	0.67411
H	0.12412	0.02631	0.19734
H	0.12284	0.92816	0.69572
H	0.62067	0.52545	0.19285
H	0.59121	0.38811	0.68292
H	0.624	0.0255	0.1941
H	0.59857	0.89279	0.69146
H	0.37885	0.00918	0.32245
H	0.37397	0.05243	0.82857
H	0.37268	0.44276	0.31972
H	0.39697	0.59184	0.84198
H	0.87134	0.96481	0.32165
H	0.87983	0.04879	0.82675
H	0.87479	0.44622	0.32098
H	0.89376	0.58352	0.84747
H	0.37138	0.27509	0.19307
H	0.3631	0.16491	0.68487
H	0.37275	0.77538	0.19391
H	0.33772	0.64118	0.69093
H	0.86926	0.27674	0.19139
H	0.86464	0.15313	0.69449
H	0.87218	0.77581	0.19437
H	0.84014	0.64111	0.68231
H	0.1233	0.20533	0.31868
H	0.12836	0.2903	0.82683
H	0.12899	0.69077	0.32153

H	0.14131	0.69583	0.84506
H	0.62185	0.19907	0.32156
H	0.62438	0.31207	0.82886
H	0.6275	0.69798	0.32249
H	0.63627	0.69151	0.8401
Ow	0.07896	0.2139	0.19086
Ow	0.08189	0.21956	0.6934
Ow	0.07866	0.73488	0.19462
Ow	0.07667	0.71358	0.68671
Ow	0.57972	0.21181	0.1909
Ow	0.58205	0.21431	0.69333
Ow	0.5744	0.71974	0.18779
Ow	0.59363	0.75746	0.71016
Ow	0.41787	0.25425	0.32445
Ow	0.41631	0.25905	0.82048
Ow	0.41023	0.7774	0.32095
Ow	0.41924	0.7485	0.82752
Ow	0.91684	0.25835	0.32246
Ow	0.91976	0.27207	0.82575
Ow	0.9164	0.77752	0.32682
Ow	0.91816	0.74796	0.82712
Ow	0.32677	0.48579	0.19401
Ow	0.32671	0.50934	0.69236
Ow	0.32827	0.96581	0.19065
Ow	0.33036	0.95744	0.6946
Ow	0.82663	0.48181	0.19184
Ow	0.82654	0.50843	0.69218
Ow	0.82908	0.96306	0.19164
Ow	0.8297	0.95985	0.69035
Ow	0.16558	0.01397	0.32192
Ow	0.16279	0.05348	0.82169
Ow	0.1671	0.49022	0.32021
Ow	0.16886	0.49471	0.826
Ow	0.66531	0.01088	0.32384
Ow	0.67149	0.01204	0.82946
Ow	0.67025	0.49096	0.32128
Ow	0.66724	0.50716	0.82941
Hw	0.12209	0.21963	0.17901
Hw	0.12606	0.21312	0.68443
Hw	0.12249	0.72991	0.1847
Hw	0.12183	0.7128	0.68438
Hw	0.62321	0.21399	0.17954
Hw	0.62661	0.21236	0.68632
Hw	0.61965	0.7197	0.18728
Hw	0.62553	0.73331	0.67776
Hw	0.37406	0.25085	0.33412

Hw	0.37232	0.2575	0.83002
Hw	0.36968	0.75946	0.33531
Hw	0.38451	0.73586	0.7905
Hw	0.87319	0.26031	0.33338
Hw	0.88476	0.23749	0.79472
Hw	0.87368	0.75618	0.33043
Hw	0.87311	0.75436	0.82948
Hw	0.37131	0.48413	0.18686
Hw	0.36071	0.50861	0.7292
Hw	0.37229	0.97223	0.18217
Hw	0.37472	0.95807	0.68713
Hw	0.87125	0.4792	0.18523
Hw	0.85913	0.50908	0.73072
Hw	0.87272	0.96605	0.18086
Hw	0.87485	0.96792	0.69058
Hw	0.12315	0.00815	0.33585
Hw	0.11988	0.03352	0.8258
Hw	0.12337	0.49277	0.33087
Hw	0.13718	0.47417	0.78613
Hw	0.62271	0.00691	0.33788
Hw	0.62616	0.01178	0.82477
Hw	0.62543	0.49699	0.32458
Hw	0.63819	0.48185	0.78768
Hw	0.06993	0.14434	0.18444
Hw	0.07101	0.16449	0.71349
Hw	0.0773	0.77961	0.24088
Hw	0.06815	0.69175	0.72968
Hw	0.56873	0.14367	0.18882
Hw	0.57883	0.19657	0.73915
Hw	0.56222	0.6634	0.20558
Hw	0.61174	0.74389	0.7555
Hw	0.42958	0.32284	0.33211
Hw	0.42066	0.30627	0.79087
Hw	0.40696	0.84729	0.32224
Hw	0.44849	0.79157	0.81208
Hw	0.93026	0.32521	0.32435
Hw	0.94158	0.3051	0.79507
Hw	0.91364	0.84621	0.32453
Hw	0.92406	0.70752	0.78059
Hw	0.32213	0.53153	0.23913
Hw	0.29001	0.52481	0.71854
Hw	0.32009	0.89654	0.18714
Hw	0.32766	0.93634	0.73927
Hw	0.82148	0.53059	0.23535
Hw	0.78937	0.5277	0.71668
Hw	0.81895	0.89406	0.18679

Hw	0.82232	0.94461	0.73605
Hw	0.17533	0.08338	0.32883
Hw	0.1649	0.12399	0.83927
Hw	0.16741	0.45136	0.27178
Hw	0.19958	0.53538	0.81033
Hw	0.67459	0.08013	0.32859
Hw	0.68623	0.043	0.79298
Hw	0.67582	0.44458	0.27671
Hw	0.69948	0.54565	0.81405
Ca	0.1861	0.02781	0.46735
Ca	0.18344	0.04702	0.96135
Ca	0.18575	0.5272	0.46296
Ca	0.18255	0.54227	0.95781
Ca	0.6849	0.02854	0.46619
Ca	0.68386	0.03987	0.95705
Ca	0.68589	0.52743	0.45929
Ca	0.68223	0.5441	0.9598
Ca	0.31014	0.44714	0.05475
Ca	0.31484	0.43394	0.5605
Ca	0.30939	0.94993	0.05342
Ca	0.31011	0.93239	0.55848
Ca	0.81029	0.44742	0.05604
Ca	0.81314	0.43122	0.5588
Ca	0.80987	0.94564	0.05163
Ca	0.81057	0.932	0.55895
Ca	0.43711	0.27774	0.46714
Ca	0.43489	0.29265	0.96181
Ca	0.43415	0.78075	0.46459
Ca	0.43074	0.79334	0.95749
Ca	0.93645	0.27518	0.4649
Ca	0.93337	0.28981	0.95813
Ca	0.9351	0.7785	0.46147
Ca	0.93387	0.7926	0.95826
Ca	0.05935	0.19752	0.05077
Ca	0.06351	0.18063	0.558
Ca	0.05943	0.69611	0.05083
Ca	0.06034	0.68112	0.55374
Ca	0.56029	0.19843	0.05332
Ca	0.56274	0.18264	0.55861
Ca	0.55914	0.69903	0.05653
Ca	0.56052	0.68058	0.54805
Cw	0.24793	0.25153	0.25963
Cw	0.25051	0.27711	0.75918
Cw	0.24584	0.75616	0.25917
Cw	0.24274	0.76659	0.76135
Cw	0.74902	0.21893	0.25527

Cw	0.74938	0.27641	0.76221
Cw	0.74548	0.75942	0.2605
Cw	0.7458	0.76506	0.75848
Cw	0.99673	0.00805	0.25994
Cw	0.99806	0.93993	0.75894
Cw	0.99744	0.50035	0.25752
Cw	0.99547	0.50336	0.75414
Cw	0.50308	0.96323	0.25492
Cw	0.49834	0.00697	0.76117
Cw	0.49853	0.46872	0.25568
Cw	0.50024	0.52671	0.75712
Ca	0.18189	0.2774	0.45888
Ca	0.17953	0.29658	0.95999
Ca	0.17826	0.77887	0.46062
Ca	0.17819	0.80041	0.96875
Ca	0.68179	0.27744	0.45851
Ca	0.67922	0.29772	0.96027
Ca	0.67919	0.78038	0.45902
Ca	0.67796	0.79697	0.96683
Ca	0.31708	0.19483	0.05454
Ca	0.31898	0.18001	0.55445
Ca	0.31339	0.69607	0.0564
Ca	0.31636	0.67823	0.55035
Ca	0.81601	0.19291	0.05379
Ca	0.81732	0.17246	0.54752
Ca	0.81422	0.69535	0.05604
Ca	0.81549	0.6771	0.54831
Ca	0.42958	0.03198	0.46598
Ca	0.42786	0.04648	0.96307
Ca	0.43042	0.52839	0.45484
Ca	0.42899	0.54881	0.97122
Ca	0.93096	0.031	0.46647
Ca	0.92833	0.0446	0.96448
Ca	0.92979	0.52769	0.45669
Ca	0.93012	0.54349	0.96628
Ca	0.06654	0.44307	0.05339
Ca	0.06633	0.42653	0.54451
Ca	0.06536	0.94769	0.05866
Ca	0.06738	0.92955	0.55808
Ca	0.5656	0.44642	0.05597
Ca	0.56827	0.42515	0.54269
Ca	0.56436	0.94536	0.05306
Ca	0.5664	0.93117	0.5565
