# Supplementary material for: Densification of the interlayer spacing governs the nanomechanical properties of calcium-silicate-hydrate
Source: Sci Rep. 2017 Sep 8;7:10986. doi: 10.1038/s41598-017-11146-8 (PMC5591233; doi:10.1038/s41598-017-11146-8)
Supplement: Supplementary file 4 — supporting information [file 41598_2017_11146_MOESM4_ESM.pdf]

Supporting Information for:

Densification of the interlayer spacing governs the nanomechanical properties of calcium-silicate-hydrate

Guoqing Geng [1,*], Rupert J. Myers [1,2,a], Mohammad Javad Abdolhosseini Qomi [3,b], Paulo J. M. Monteiro[1,4,**]

[1] Department of Civil and Environmental Engineering, University of California, Berkeley, California 94720, United States

[2] School of Forestry & Environmental Studies, Yale University, New Haven, Connecticut 06511, United States

[3] Department of Civil and Environmental Engineering, University of California, Irvine, California 92697, United States

[4] Material Science Division, Lawrence Berkeley National Laboratory, Berkeley, California 94720, United States

[*] Corresponding author: E-mail: guoqinggeng1989@gmail.com; Tel. +1(510)5902744; Postal address: 115 Davis Hall, University of California, Berkeley, California 94720, United States.

[**] Corresponding author: E-mail: monteiro@berkeley.edu; Tel. +1(510)6438251; Postal address: 725 Davis Hall, University of California, Berkeley, California 94720, United States.

[a] rupert.myers@gmail.com, [b] mjaq@uci.edu

I. HIGH PRESSURE X-RAY DIFFRACTION

The HP-XRD experiment was conducted at beamline 12.2.2 of the Advanced Light Source (ALS), Lawrence Berkeley National Laboratory (LBNL). Stainless steel gaskets and diamond anvils of culet diameter ~300 μm were used in an axial Merrell-Bassett cell. The gaskets were pre-indented using diamond anvils (~300 μm diameter) before laser drilling holes through the centers of the indented regions to create cylindrical chambers of diameter 150 μm and height ~100 μm (one for each gasket). Small amounts of ruby powder (α-Al_2O_3 doped with 0.05 wt.% Cr^{3+}) were mixed with the samples in the chambers, and was used as the pressure calibrant. The chamber was then filled with methanol-ethanol (volumetric ration 4:1), and then immediately closed using the diamond culets. This methanol-ethanol solution intrudes pores of ~1 nm in diameter[1,2] and therefore generates hydrostatic pressure on porosity-free nanocrystalline C-S-H. Hydrostatic pressure up to ~10 GPa, with step size 1-2 GPa, was generated by applying load on the diamond anvils in the direction parallel to the incident beam path. The pressure was calibrated using the ruby fluorescence signal at each step[3]. HP-XRD patterns were recorded on a MAR345 detector, with the center positions and sample-to-detector distance calibrated by LaB_6. The raw diffraction data were integrated using the Dioptas software[4]. The incident beam energy was set to ~25 keV and the wavelength was calibrated to be 0.49755 Å. Rietveld refinement was performed using the MAUD software[5].

The change in molar volume of the solid phase, induced by the applied hydrostatic pressure, is linked to the bulk modulus by the second order BM-EoS[6] (equation 1):

$$P = \frac{3}{2} K_0 [(1-\varepsilon_V)^{-\frac{7}{3}} - (1-\varepsilon_V)^{-\frac{5}{3}}] \qquad (1)$$

where the volumetric strain ε_V =1-V/V_0; V_0 is the unit cell volume at ambient pressure; V is the unit cell volume under applied hydrostatic pressure P; and K_0 is the ambient bulk modulus. This equation is valid when the first pressure derivative of K_0 = 4, which is often assumed for clay mineral in order to more confidently fit K_0 [7,8].

II. THE 2D RAW DIFFRACTION DATA OF 1.0CSH AND 1.3CSH AT AMBIENT PRESSURE

The raw diffraction images of 1.0CSH and 1.3CSH at ambient pressure are shown in Fig. S1. Similar as that of 0.8CSH, continuous diffraction rings are observed to be consistent with the reported diffractions of C-S-H(I). Discrete diffraction spots are assigned to ruby (Cr^{3+}-doped α-Al_2O_3, used as pressure calibrant) and oxides of the steel gasket.

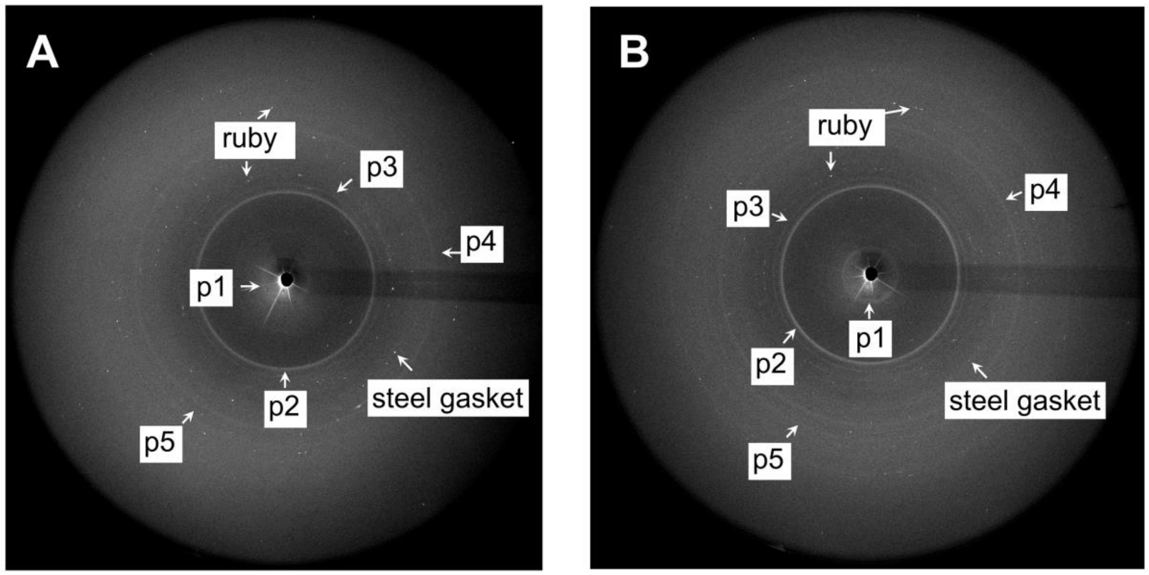

Fig. S1. The 2D raw diffraction data of 1.0CSH (*A*) and 1.3CSH (*B*) at ambient pressure. Ruby and steel gasket yield multiple diffraction spots, and the arrows only point out a few of them as demonstration.

III. DETERMINING THE ERROR OF THE REFINED LATTICE PARAMETERS

The lattice parameters *a*, *b*, and *c* are refined with the *MAUD* package[20], using the integrated XRD diffractogram at each pressure value. To address the anisotropic crystallite size effect, the "anisotropic – no rules" line broadening models was used, where the crystallite sizes, anisotropic micro-strains and the lattice parameters are readily defined.[9] The uncertainties of refining *a*, *b*, and *c* are conservatively determined as 0.01 Å, 0.01 Å and 0.1 Å. Changing the lattice parameters by such values results in a noticeable peak shifting, as shown in an example in Fig. S2.

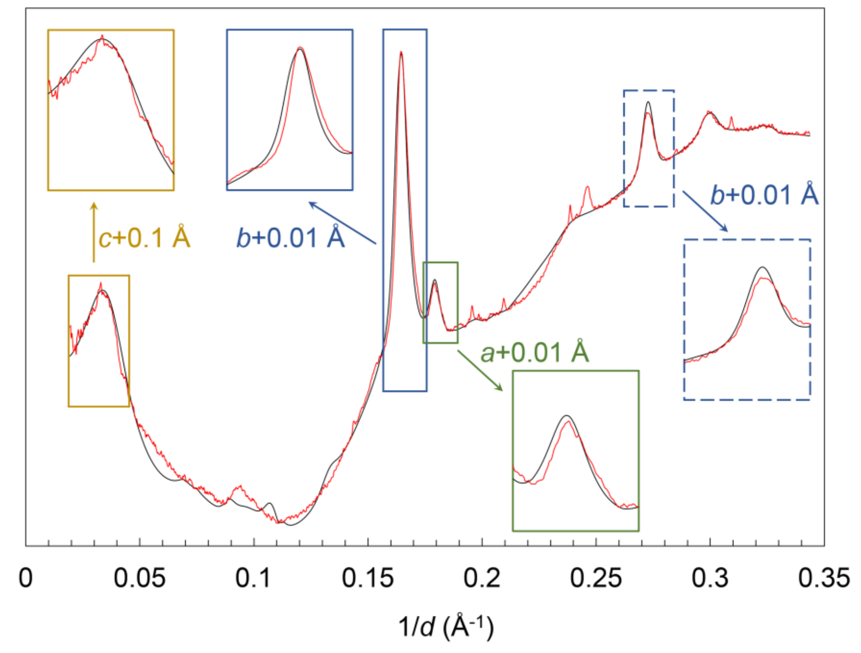

Fig. S2. Determining the uncertainties of refining the lattice parameters. Red and black curves are the calculated and experimental diffractograms, respectively, of 0.8CSH at 2.14 GPa. The insets are the local peak shifting resulting from changes of the lattice parameters with the indicated increments, compared with the best fitting results.

IV. REFINING THE LATTICE PARAMETERS AT ELEVATED PRESSURE

As shown in Fig. S3 *A*-*C*, all of the diffraction peaks of nanocrystalline C-S-H shift to smaller *d*-spacings under increasing hydrostatic pressure, and return to their original positions upon unloading to ambient pressure, indicating that the samples undergo reversible compression. The consistent peak shift trends and the absence of peak splitting or new peak generation imply that reconstructive changes of the C-S-H crystal structure do not occur. We observe a pressure-induced strain broadening which becomes increasingly significant at higher hydrostatic pressure,[8] such that we are unable to resolve p1 above 7.10 and 8.06 GPa for the 0.8CSH and 1.0CSH samples, respectively. This effect limits the refinement of parameter *c* at higher pressures, although we can confidently refine parameters *a* and *b* up to ~10 GPa. Refinement of C-S-H lattice parameters at each hydrostatic pressure value yields the results shown in Fig. S3 *D*-*F*.

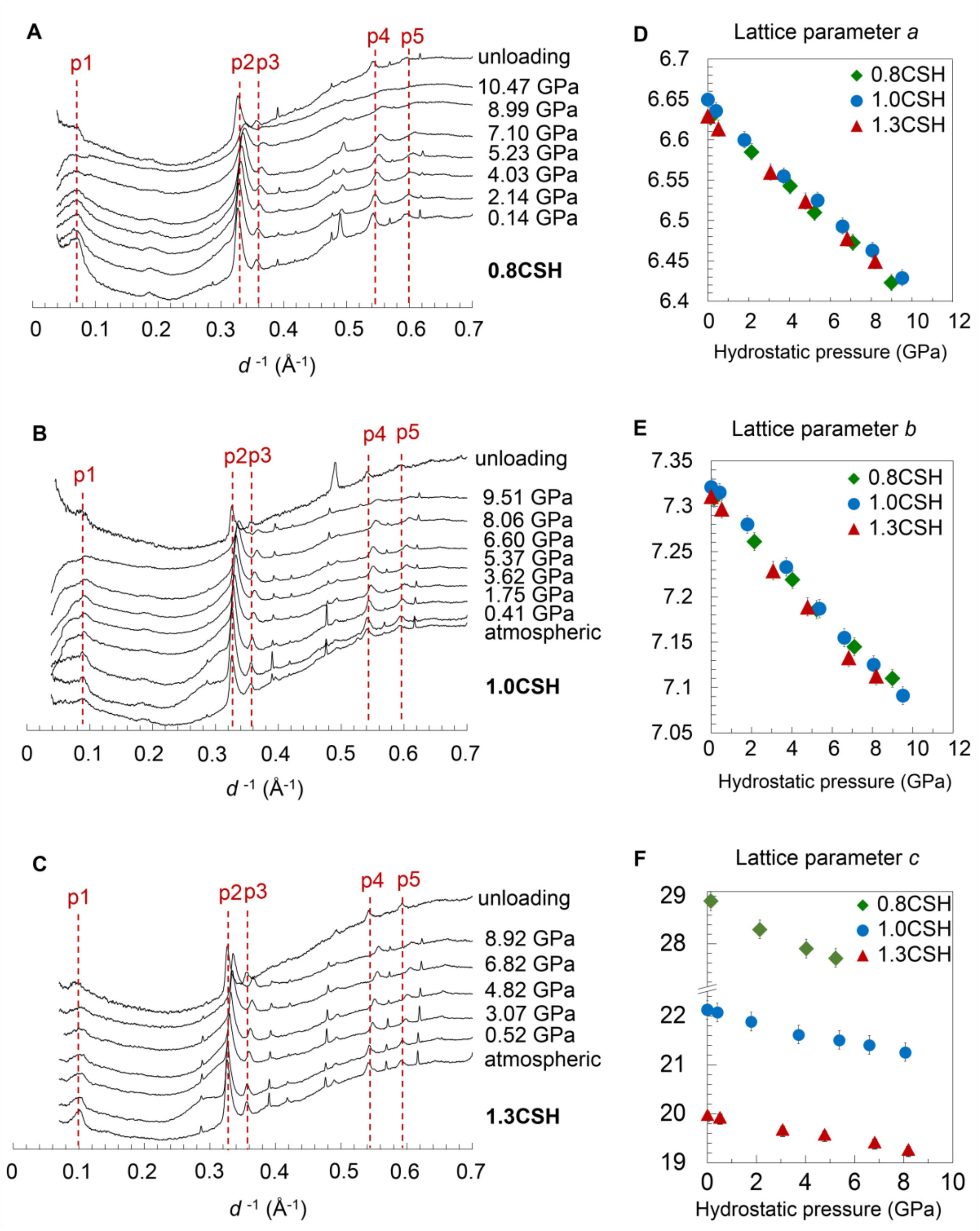

Fig. S3. X-ray diffraction data and lattice parameters as functions of the applied hydrostatic pressure: Diffractograms of 0.8CSH (*A*), 1.0CSH (*B*), 1.3CSH (*C*); pressure-induced change of lattice parameters *a* (*D*), *b* (*E*), and *c* (*F*). The lengths of the error bars for the lattice parameters *a*, *b*, and *c* are 0.01, 0.01, and 0.1 Å, respectively. The error in pressure calibration is ~0.2 GPa.

Note that the 14 Å tobermorite structure (used as the starting configuration of the refinement), is monoclinic, with $\alpha=\beta=90°$ and $\gamma=123.3°$. The variation in the lattice parameter γ of ±0.1° was previously reported for 14 Å tobermorite at pressures up to ~5 GPa,[8] causing a volumetric strain of ≤ ±0.001. This small volumetric strain has a negligible effect on the determination of the bulk modulus. Therefore, during the refinement procedure at elevated hydrostatic pressures, we fix γ to the refined values obtained at the ambient condition, i.e., 122.6°, 122.8°, and 122.5° for 0.8CSH, 1.0CSH, and 1.3CSH, respectively. The refined lattice parameters at ambient pressure are given in Table S1.

Table S1. The refined lattice parameters at ambient pressure.

	0.8CSH	1.0CSH	1.3CSH
Ca/Si	0.8	1.0	1.3
a (Å)	6.63±0.01	6.65±0.01	6.63±0.01
b (Å)	7.31±0.01	7.32±0.01	7.31±0.01
c (Å)	28.9±0.1	22.1±0.1	19.9±0.1
α	90°	90°	90°
β	90°	90°	90°
γ	122.6°±0.5	122.8°±0.1°	122.5°±0.1°
V (Å^3)	1180±7	904±7	813±7

V. ATOMISTIC SIMULATIONS

In this work, we use transferrable CSH-FF potential to describe interatomic interactions.[10] Starting from the ambient pressure C-S-H models, we subsequently increased the external pressure up to 8 GPa with intervals of 0.1 GPa and relaxed both atomic positions and lattice vectors to locally minimize the enthalpy. We perform three loading-unloading sequences to ensure that our pressure-volume data is unaffected by the

positions and local orientations of the nano-confined water molecules in the interlayer space. The pair distribution function, atomic distances and angles are calculated on the fully relaxed structures. Structural relaxation is performed by enthalpy minimization technique with a convergence norm tolerance of 10^{-8}, while enforcing rational functional optimization below a norm of 10^{-4} to avoid unstable configurations. For this study, we use the GULP package along with a suite of pre- and post-processing tools to facilitate finite difference-based pressure-volumetric strain equation of state calculations.[11]

The computational strategy used here, aiming to vary Ca/Si systematically and explore resulting properties, is a combination of several classical atomistic simulation techniques. These methods are deployed at different stages of the model preparation. The complex hydrate materials have different types of atoms with varying chemical environments, which is discussed in details in following subsections. These simulations, which have classical nature, are performed using CSH-FF potentials.[10] The main chemical elements in C-S-H are Si, Ca, O and H atoms. These atoms adopt different characteristics depending on their local chemical environments. To appropriately address such variations, classical potentials utilize different labels for a given element to describe these chemical environments. Due to charge compensation, the O-H bonding would be different inside a water molecule and a hydroxyl group. Therefore, for classical simulations using CSH-FF potential, one needs the prior knowledge about the local atomic environments in the molecular structure of material. More specifically, CSH-FF uses nine labels to describe the four types of elements in C-S-H. Our convention for these nine labels is as follows: Ca (intralayer calcium), Cw (interlayer calcium), Si (silicon), Oh (oxygen in hydroxyl group), H (hydrogen in hydroxyl group), Ob (bridging oxygen in silica chain), Ow (oxygen in water molecule), Hw

(hydrogen in water molecule), O (oxygen other than Ow, Oh and Ob). The partial charges of all species are provided in Table S2. The partial charges of all species are constant except oxygen atoms. In fact, the partial charges of oxygen species in calcium-silicate layers are set to vary in order to ensure charge-neutrality within numerical samples. A comprehensive sensitivity analysis on the elastic properties is carefully performed with different charge-balancing schemes. Due to the small charge-imbalance in the samples, charge-neutralization schemes do not strongly affect the elastic properties. In this work, the excess charge is equally distributed among O, Ob and Oh atoms.

Table S2. Partial Charges in CSH-FF potential.

Species	Charge	Species	Charge
Si	1.722357	H	0.425
Ca	1.435466	O	[-1.15,-1.26]
Cw	1.705529	Oh	[-0.93,-0.97]
Ow	-0.82	Ob	[-1.01,-1.13]
Hw	0.41		

The CSH-FF interatomic/intermolecular potential is a core-only model for hydrated calcium-silicates, in which atoms are described as interacting points through Columbic and short-range potential functions. The total interatomic potential for CSH-FF potential reads:

$$E_{tot}^{CSHFF} = E_{Col} + E_{lj} + E_{radial} + E_{angular} \quad (1)$$

where E_{Col}, E_{lj}, E_{radial}, and $E_{angular}$ are the electrostatic, short-range Van der Waals in the form of Lennard-Jones potential, bond stretching and bond bending contributions to the potential energy, respectively. CSH-FF potential is a customized force field for hydrated calcium silicates, and it is not transferable to other oxides with different chemical environments. Therefore, CSH-FF can be seen as an improved version of the generic

ClayFF potential (originally developed for structural properties of clay minerals[12]) for hydrated calcium-silicates such as cement hydrates. To describe the molecular structure of water and its interaction with other water molecules, both CSH-FF and Clay-FF potential models are built around the flexible SPC model for water.[13] In particular, CSH-FF was designed to reproduce both structural and elastic data of tobermorite minerals obtained from ab initio calculations.

In order to extend the predictability of the CSH-FF model, the latter was slightly modified to include a couple of new features. Primarily to reduce the computational costs incurred by the calculation of long-range forces using Ewald summation technique, the columbic interactions are calculated using Wolf method.[14] While Ewald summation scales with O($N^{3/2}$), Wolf summation scales with O(N). To apply a uniform cut-off radius in the simulation box for both long- and short-range interactions, R_c is set to 12Å. The Wolf damping factor, η, is set to 0.25 in all simulations. The second modification in the CSH-FF potential pertains to incorporation of hydroxyl groups. The force field parameters for hydroxyl groups are included in the extended form of the CSH-FF potential.

The short-range interactions in CSH-FF and ClayFF force fields are described via Lennard-Jones potential:

$$E_{lj} = 4\epsilon \sum_i \sum_{j>i} \left[\left(\frac{\sigma}{r_{ij}}\right)^{12} - \left(\frac{\sigma}{r_{ij}}\right)^{6} \right] \quad (2)$$

where σ and ϵ have the dimensions of distance and energy, respectively. These parameters are provided for short-range interactions between pairs of elements in the model. The potential parameters are provided in Table S3.

Table S3. Lennard-Jones potential parameters used in CSH-FF potential.

$spec_i$	$spec_j$	ϵ (kcal/mol)	σ (Å)	$spec_i$	$spec_j$	ϵ (kcal/mol)	σ (Å)
O	Ca	8.694E-4	4.365	Oh	Oh	6.180E-2	3.448
Ca	Ob	7.057E-4	5.452	O	Ob	4.543E-2	3.626
Ca	Oh	8.694E-4	4.365	O	Oh	4.543E-2	3.626
O	Cw	1.460E-3	4.365	O	Ob	6.180E-2	3.448
Cw	Ob	1.040E-3	4.446	Ca	Ow	8.760E-4	4.365
Cw	Oh	1.040E-3	4.446	Cw	Ow	6.042E-4	4.472
O	Si	5.604E-4	3.270	O	Ow	5.258E-3	4.241
Si	Ob	5.950E-4	3.261	Ow	Ob	8.717E-1	2.895
Si	Oh	5.950E-4	3.261	Ow	Oh	8.717E-1	2.895
Si	Ow	5.950E-4	3.235	Ow	Ow	1.540E-1	3.163
Ob	Ob	6.180E-02	3.448	O	O	1.243E+0	2.735

Similar to ClayFF potential, the hydrogen species do not have short-range interactions. The radial and angular excursions in water molecules and hydroxyl groups are described via simple harmonic potentials:

$$E_{radial} = \sum_{i=1}^{N_{OH}} \frac{1}{2} k_r \left(r_{ij} - r_0\right)^2 \quad (3)$$

$$E_{angular} = \sum_{i=1}^{N_{H_2O}} \frac{1}{2} k_\theta \left(\theta_{ij} - \theta_0\right)^2 \quad (4)$$

where k_r and k_θ are radial and angular stiffness, respectively. Parameters r_0 and θ_0 denote the equilibrium hydroxyl bond length and Hw-Ow-Hw angle in water molecules, respectively. N_{OH} and N_{H2O} are the total number of O-H bonds in hydroxyl groups and water molecules and total number of water molecules, respectively. The radial and angular constants are provided in the Table S4 and S5.

Table S4. Radial interaction Constants in CSH-FF potential.

$spec_i$	$spec_j$	k_r (Kcal/mol/Å^2)	r_0 (Å)
Hw	Ow	554.13	1
H	Oh	554.135	1

Table S5. Angular interaction within water molecules in CSH-FF potential.

$spec_i$	$spec_j$	$spec_k$	k_θ (Kcal/mol/ θ^2)	θ_0
Hw	Ow	Hw	45.770	109.47

VI. INITIAL ATOM POSITIONS FOR MOLECULAR SIMULATION

The initial atom positions for calculation are determined in the following steps:

Step 1, determining the starting configuration from published tobermorite structure, which are generally classified into three groups according to the basal spacing, i.e. 9.3 Å, 11.3 Å, 14.0 Å tobermorite.[15-19] We start from tobermorite structures with the best match to the measured crystal-chemical features of the C-S-H samples. The 0.8CSH has basal spacing is ~14.42 Å at ambient pressure, and therefore the 14 Å tobermorite structure was chosen as the starting configuration. Similarly the 9 Å tobermorite structure (basal spacing ~9.57 Å) was used for the 1.3CSH (basal spacing ~9.97 Å at ambient pressure). The ambient pressure basal spacing of 1.0CSH is ~11.07 Å, close to that of 11 Å tobermorite (~11.24 Å) of both normal and anomalous polymorph. However, the NMR data of 1.0CSH confirm no dreierketten chain crosslinking, in contrast to these two 11 Å tobermorite structures. Therefore, we also used the 14 Å tobermorite structure as the starting configuration for 0.8CSH.

Step 2, modifying the dreierketten chain. To guarantee similar dimensions along each axis, 2×2×1 supercells were constructed for 0.8CSH and 1.0CSH, and a 2×2×2 supercell

was constructed for 1.3CSH, using the starting configurations determined in step 1. The size of the supercell also enables adjusting the mean dreierketten-chain length (MCL) to the measured values of all samples. The measured MCL are 19, 5 and 2 with increasing Ca/Si.[20] For non-crosslinked CSH, the MCL is related to the bridging site omission percentage (α) by equation (5):

$$MCL = (3-\alpha) / \alpha \qquad (5)$$

The omission percentages are back-calculated as 15%, 50% and 100% for samples with increasing Ca/Si. Therefore the corresponding amount of bridging site Si was removed in the starting configuration. The vacancies of bridging site are assumed not agglomerating but rather in homogeneous distribution in the models of 0.8CSH and 1.0CSH, as the agglomeration of the same type of defects is proven not energetically favored.[21] Note that the 1.3CSH has a completely dimeric structure.

Step 3, determining the amount of interlayer contents. The intralayer CaO_7 double sheets were kept unchanged. The existing interlayer Ca atoms in the starting crystal structure of tobermorite were also conserved, and a certain number of extra Ca atoms were placed at a position with the biggest distance to existing interlayer Ca, so that the total Ca/Si molar ratios matches the measure bulk Ca/Si ratios.

The conventional thermal gravity data exhibit significant deviations in determining the amount of water in the interlayer of C-S-H.[22] It is our major purpose to study the correlation between the mechanical responses of C-S-H with the interlayer spacing. Thus determining the amount of interlayer water requires extra care here, as it strongly correlates with the interlayer spacing.[23] We used difference amount of interlayer water as input for molecular simulation and, from the relaxed structure, determine the amount of interlayer water that

outputs an interlayer spacing within 2% deviation from the XRD refined value at ambient pressure.

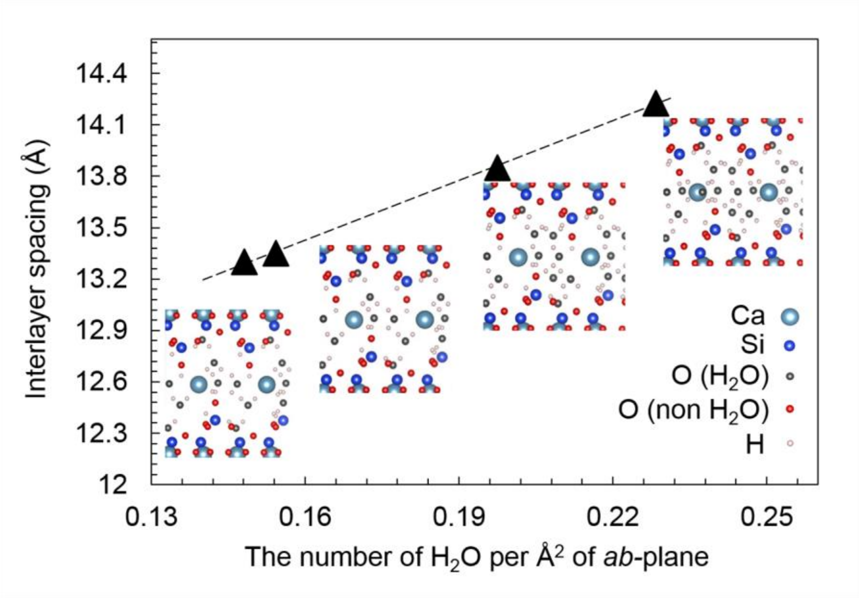

Fig. S4. GULP relaxed basal spacing of 0.8CSH as a function of the amount of interlayer water molecules per $Å^2$ of the *ab*-plane per interlayer. The dash line is to eye-guide the trend.

As shown in Fig S4, the basal spacing increases roughly linearly with increasing the amount of interlayer water per $Å^2$ of *ab*-plane. We adopt the 0.23 H_2O / $Å^2$ per interlayer as the value used in the initial structure for 0.8CSH, since the relaxed basal spacing (~14.23 Å) is close enough (within 2% deviation) to the XRD measured value (~14.42 Å). No special attempt was made to match the exact value from XRD measurement. Similarly, we determine the number density of interlayer water in 1.0CSH to be 0.15 H_2O / $Å^2$ per interlayer, and in 1.3CSH to be 0.05 H_2O / $Å^2$ per interlayer, corresponding to relaxed basal spacings of ~11.30 Å and ~9.99 Å, respectively. The measured basal spacings are ~11.07 Å for 1.0CSH and ~9.97 Å for 1.3CSH, both within 2% deviation compared with the relaxed model.

Step 4, to balance the sampling size and computation efficiency, we use 2×2×1 supercells for 0.8CSH and 1.0CSH, and 2×2×2 supercell for 1.3CSH (after modification of previous steps) as the starting model of calculation. To insure smooth loading curve, the models undergoes two simulative loading-unloading cycles, up to 8 GPa hydrostatically. The obtained atom positions at 0 GPa are then used as the initial models for simulation. They are listed in Table S6 (0.8CSH), S7 (1.0CSH) and S8 (1.3CSH) in the form of fractional coordinates (the tables are too long and are available as individual supplemental materials). The relaxed configurations are plotted in Fig S5. The amount of species in each model is given in Table S9.

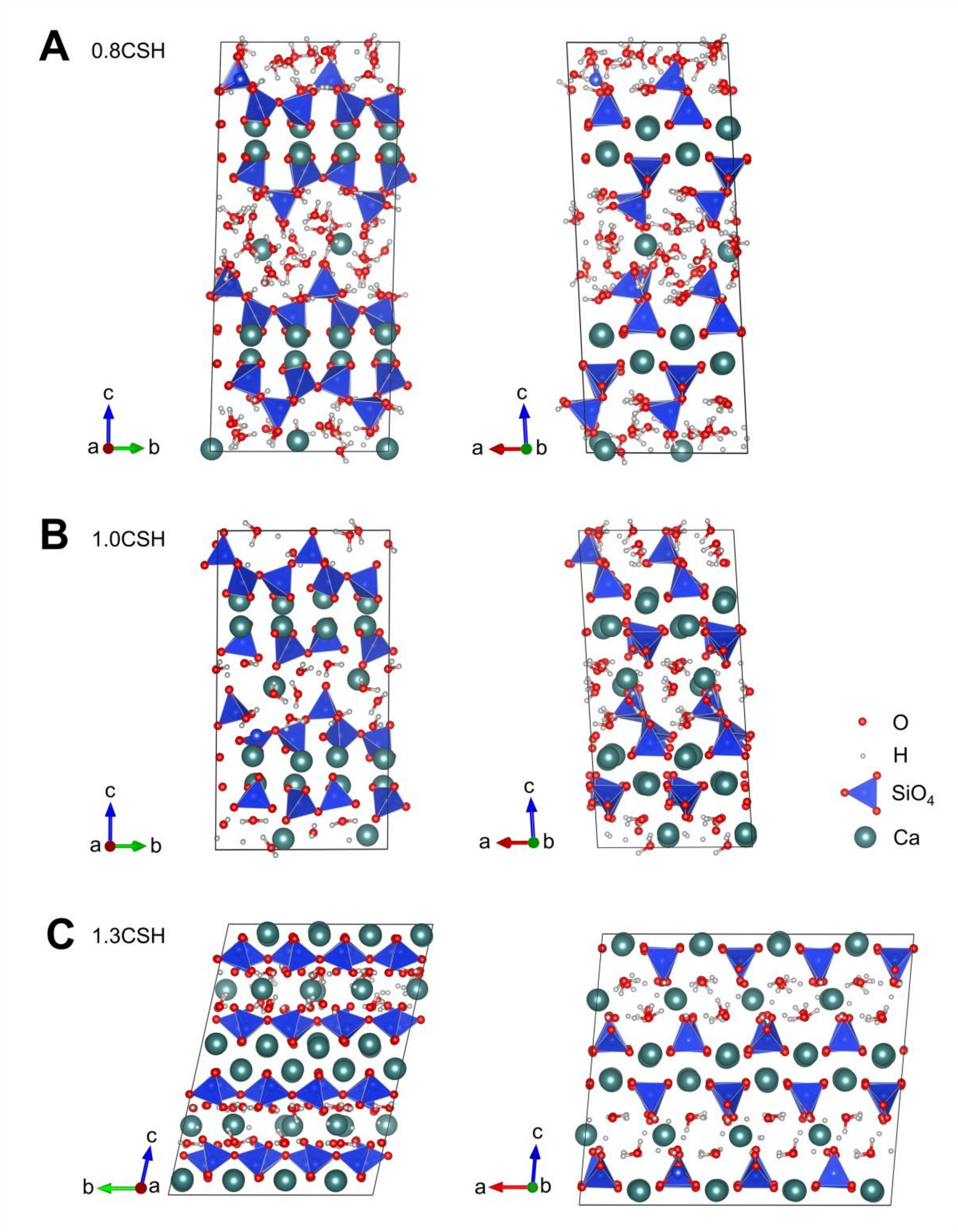

Fig. S5. The ambient pressure configurations of C-S-H models: (*A*) 0.8CSH, (*B*) 1.0CSH and (*C*) 1.3CSH.

Table S9. The amount of different species in each model: Ca and Cw represent Ca in the interlayer and intralayer region, respectively; Oh and Ob represent hydroxyl group and bridging oxygen, respectively. The rest oxygens (non-bridging and not connected to H) are counted as O.

Species	0.8CSH	1.0CSH	1.3CSH
Si	46	40	64
Ca_inter	32	32	64
Ca_intra	5	8	16
Water	77	40	32
Oh	26	16	96
Ob	44	32	32
O	72	80	128

VII. ALGORITHM OF CALCULATING THE PAIR DISTRIBUTION FUNCTION (PDF)

A number of different formalisms for PDFs exist in the literature. Keen performed an extensive survey of these and we follow his recommendations.[24] The calculation of Pair Distribution Functions is in accordance with the theory of Chung and Thorpe.[25] It makes use of phonon information calculated within GULP,[11] for an optimized structure, using a Monkhorst-Pack grid. Chung and Thorpe state that the probability of finding a pair of atoms *i* and *j*, with position $\boldsymbol{r}_i$ and $\boldsymbol{r}_j$ respectively, at position r is given by

$$\rho_{ij}(\boldsymbol{r}) = \langle \delta\left(\boldsymbol{r} - \left(\boldsymbol{r}_j - \boldsymbol{r}_i\right)\right)\rangle \quad (6)$$

where <…> is the statistical average implying both configurational and thermal averages. To account for thermal motion, Chung and Thorpe demonstrated that, within the harmonic approximation, the Debye-Waller theorem can be used to justify the use of a series of

weighted Gaussian peaks $\rho_{ij}(\boldsymbol{r})$, centered at $\boldsymbol{r}_{ij}$ to be the unit vector between atoms *i* and *j*, and $\boldsymbol{u}_{ij} = \boldsymbol{u}_j - \boldsymbol{u}_i$, where $\boldsymbol{u}_i$ is the displacement of atom *i*. then the width is given by

$$\sigma_{ij} = \langle \boldsymbol{u}_{ij} . \boldsymbol{r}_{ij} \rangle \tag{7}$$

Therefore, we can express $\rho_{ij}(\boldsymbol{r})$ as the sum of the Gaussian peaks for each pair in the main unit cell and its periodic images, as follows:

$$\rho_{ij}(r) = \sum_i \sum_j \sum_l \frac{1}{\sqrt{2\pi\sigma_{i0jl}^2}} \exp\left[\frac{|r_{i0jl}| - r}{2\sigma_{i0jl}^2}\right] \tag{8}$$

where subscripts *0* and *l* pertain to the unit cell and its *l*th periodic image. The pair distribution function is subsequently calculated following:

$$g_{ij}(r) = 4\pi r\left[\rho_{ij}(r) - \rho_0\right] \tag{9}$$

where ρ_0 is the average number density, $\rho_0 = n/V_{unitcel}$.

References

1 Jennings, H. M., Thomas, J. J., Gevrenov, J. S., Constantinides, G. & Ulm, F. J. A multi-technique investigation of the nanoporosity of cement paste. *Cem. Concr. Res.* **37**, 329-336 (2007).

2 Juenger, M. C. G. & Jennings, H.M. New insights into the effects of sugar on the hydration and microstructure of cement pastes. *Cem. Concr. Res.* **32**, 393-399 (2002).

3 Piermarini, G. J., Block, S., Barnett, J. D. & Forman, R. A. Calibration of the pressure dependence of the R1 ruby fluorescence line to 195 kbar. *J. Appl. Phys.* **46**, 2774 (1975).

4 Prescher C. & Prakapenka, V. B. DIOPTAS: a program for reduction of two-dimensional X-ray diffraction data and data exploration. *High Pressure Res.* **35**, 223 (2015).

5 Lutterotti, L., Matthies, S. & Wenk. H. R. MAUD: a friendly Java program for material analysis using diffraction. *IUCr: Newsletter of the CPD.* **21,** 14-15 (1999).

6 Birctt, F. Elasticity and constitution of the earth's interior. *J. Geophys. Res.* **57** (1952).

7 Prewitt C. T. & Downs, R. T. High-pressure crystal chemistry. *Rev. Mineral.* **37**, 284-318 (1998).

8 Oh, J. E., Clark, S. M., Wenk, H. R. & Monteiro, P. J. M. Experimental determination of bulk modulus of 14Å tobermorite using high pressure synchrotron X-ray diffraction. *Cem. Concr. Res.* **42**, 397-403 (2012).

9 Lutterotti, L. & Scardi, P. Profile fitting by the interference function. *Adv. X-Ray Anal.* **35,** 577-584 (1991).

10 Shahsavari, R., Pellenq, R. J. M. and Ulm, F. J. Empirical force fields for complex hydrated calcio-silicate layered materials. *Phys. Chem. Chem. Phys*. 13, 1002-1011 (2011).

11 Gale, J. D. GULP: A computer program for the symmetry-adapted simulation of solids. *J. Chem. Soc., Faraday Trans.* **93,** 629-637 (1997).

12 Cygan, R. T., Liang, J. J. & Kalinichev, A. G. Molecular models of hydroxide, oxyhydroxide, and clay phases and the development of a general force field. *J. Phy. Chem. B*, **108,** 1255-1266 (2004).

13 Wu, Y., Tepper, H. L. & Voth, G. A. Flexible simple point-charge water model with improved liquid-state properties. *J. Chem. Phy.* **124,** 024503 (2006).

14 Wolf, D., Keblinski, P., Phillpot, S. R. & Eggebrecht, J. Exact method for the simulation of Coulombic systems by spherically truncated, pairwise r^{-1} summation. *J. Chem. Phy*. **110,** 8254-8282 (1999).

15 Merlino, S., Bonaccorsi, E. & Armbruster, T. The real structures of clinotobermorite and tobermorite 9 Å OD character, polytypes, and structural relationships. *Eur. J. Mineral.* **12,** 411-429 (2000).

16 Hamid, S. A. The crystal structure of the 11 Å natural tobermorite $Ca_{2.25}[Si_3O_{7.5}(OH)_{1.5}]\cdot H_2O$. *Z. Kristallogr. Cryst. Mater.* **154,** 189-198 (1981).

17 Merlino, S., Bonaccorsi, E. & Armbruster, T. Tobermorites: their real structure and order-disorder (OD) character. *Am. Mineral.* **84,** 1613-1621 (1999).

18 Merlino, S., Bonaccorsi, E. & Armbruster, T. The real structure of tobermorite 11Å normal and anomalous forms, OD character and polytypic modifications. *Eur. J. Mineral.* **13,** 577-590 (2001).

19 Bonaccorsi, E., Merlino, S. & Kampf, A. R. The crystal structure of tobermorite 14 Å (Plombierite), a C-S-H phase. *J. Am. Ceram. Soc.* **88,** 505–512 (2005).

20 L'Hôpital, E., Lothenbach, B., Kulik, D. A. & Scrivener, K. Influence of calcium to silica ratio on aluminium uptake in calcium silicate hydrate. *Cem. Concr. Res.* **85,** 111-121 (2016).

21 Pegado, L., Labbez, C. & Churakov, S.V. Mechanism of aluminium incorporation into C–S–H from ab initio calculations. *J. Mat. Chem. A* **2,** 3477-3483 (2014).

22 Richardson, I. G. Model structures for C-(A)-S-H (I). *Acta Crystallogr. Sect. B.* **70,** 903-923 (2014).

23 Alizadeh, R., Beaudoin, J. J. and Raki, L. C–S–H (I)—A Nanostructural Model for the Removal of Water from Hydrated Cement Paste? *J. Am. Ceram. Soc.* **90,** 670-672 (2007).

24 Keen, D. A. A comparison of various commonly used correlation functions for describing total scattering. *J. App. Cryst.* **34,** 172-177 (2001).

25 Chung, J. S. & Thorpe, M. F. Local atomic structure of semiconductor alloys using pair distribution functions. *Phy. Rev. B*, **55,** 1545 (1997).
